# Three-dimensional structure discrepancy between HLA alleles for effective prediction of aGVHD severity and optimal selection of recipient-donor pairs: a proof-of-concept study

**DOI:** 10.18632/oncotarget.5378

**Published:** 2015-10-16

**Authors:** Hongxing Han, Fang Yuan, Yuying Sun, Jinfeng Liu, Shuguang Liu, Yuan Luo, Fei Liang, Nan Liu, Juan Long, Xiao Zhao, Fanhua Kong, Yongzhi Xi

**Affiliations:** ^1^ Department of Immunology and National Center for Biomedicine Analysis, Beijing 307 Hospital Affiliated to Academy of Military Medical Sciences, Beijing, China

**Keywords:** acute graft-versus-host disease, HLA alleles, three-dimensional structure discrepancy, optimizing recipient-donor selection, allogeneic CTLs reaction

## Abstract

The optimal selection of recipient-donor pair and accurate prediction of acute graft-versus-host disease (aGVHD) severity are always the two most crucial works in allogeneic hematopoietic stem cell transplantation (allo-HSCT), which currently rests mostly with HLA compatibility, the most polymorphic loci in the human genome, in clinic. Thus, there is an urgent need for a rapid and reliable quantitative system for optimal recipient-donor pairs selection and accurate prediction of aGVHD severity prior to allo-HSCT. For these reasons, we have developed a new selection/prediction system for optimal recipient-donor selection and effective prediction of aGVHD severity based on HLA three-dimensional (3D) structure modeling (HLA-TDSM) discrepancy, and applied this system in a pilot randomized clinical allo-HSCT study. The 37 patient-donor pairs in the study were typed at low- and high-resolution levels for HLA-A/-B/-DRB1/-DQB1 loci. HLA-TDSM system covering the 10000 alleles in HLA class I and II consists of the revised local and coordinate root-mean-square deviation (RMSD) values for each locus. Its accuracy and reliability were confirmed using stably transfected Hmy2.CIR–HLA-B cells, TCR Vβ gene scan, and antigen-specific alloreactive cytotoxic lymphocytes. Based on the preliminary results, we theoretically defined all HLA acceptable versus unacceptable mismatched alleles. More importantly, HLA-TDSM enabled a successful retrospective verification and prospective prediction for aGVHD severity in a pilot randomized clinical allo-HSCT study of 32 recipient-donor transplant pairs. There was a strong direct correlation between single/total revised RMSD and aGVHD severity (92% in retrospective group vs 95% in prospective group). These results seem to be closely related to the 3D structure discrepancy of mismatched HLA-alleles, but not the number or loci of mismatched HLA-alleles. Our data first provide the proof-of-concept that HLA-TDSM is essential for optimal selection of recipient-donor pairs and effective prediction of aGVHD severity before allo-HSCT.

## INTRODUCTION

Acute graft-versus-host disease (aGVHD) incidence and severity depend on several known objective risk factors [1, 2], including a wide range of transplant-related immune gene polymorphisms, such as HLA, minor histocompatibility antigen (mHA), Th1-Th2-Th3-cytokine, and Killer cell immunoglobulin-like receptors (KIR) [3–7]. Among these, HLA, an innate “transplantation barrier”, has been universally accepted as the primary factor affecting aGVHD, particularly with the increasing use of HLA allele-mismatched donors [3, 8–10]. This is because HLA mismatching affect aGVHD development owing to their fundamental roles in T cell activation, regulatory T cell inactivation, and the allo- and auto-response induction [3, 8–10]. Furthermore, aGVHD severity is likely related to the mismatched HLA loci, the total number of mismatched HLA-alleles, high-risk HLA-allele mismatched combinations, or even several key amino acid substitutions on specific positions in HLA class I, though there exists considerable controversy over these issues [8, 11–14]. More importantly, complete matching for both HLA class I and II alleles can indeed significantly decrease aGVHD severity, transplant-related mortality (TRM), and graft rejection, even though the actual requirement with regard to HLA compatibility and the relative importance of matching individual HLA alleles in allogeneic hematopoietic stem cell transplantation (allo-HSCT) have not been clearly established [9, 11, 15–18].

The problem exists in that the chance of finding an individual with an identical HLA genotype, either a relative or an unrelated donor, is low for the majority of patients in need of allo-HSCT [9, 11, 19, 20]. Thus, since high-resolution typing has been accepted as a standard confirmatory technique, many transplant centers have begun to use related and unrelated donors having 1 or more mismatched alleles at HLA-A/-B/-DRB1 loci, including HLA-Cw/-DQB1/-DPB1 loci [21–23]. Subjectively speaking, the occurrence of aGVHD in these transplanted patients will undoubtedly be more frequent and more severe. But are not all the cases in the most recent transplant studies including the National Marrow Donor Program (NMDP), Japan Marrow Donor Program (JMDP), Bone Marrow Donor Worldwide (BMDW) and Fred Hutchinson Cancer Research Center (FHCRC), etc., in which these centers used either “subset analysis” or/and “multivariate modeling” to retrospectively evaluate the impact of HLA-allele matching and mismatching on aGVHD incidence and severity, TRM and so on over 10000 transplanted donor-recipient pairs [12–14, 19, 20, 24–26]. These results suggest that allo-HSCT with mismatched HLA alleles, so-called acceptable/permissible/beneficial mismatched alleles/antigens [25, 27, 28], has therapeutic potential; however, there are insufficient experimental and clinical data to support this idea as a clinical standard practice.

Based on the overwhelming evidence that (1) the biological functions of HLA molecules are determined largely by their three-dimensional (3D) structures; and (2) 3D structural differences in peptide-binding and T-cell receptor (TCR) interaction sites can significantly alter the immunogenicity of mismatched HLA molecules, which may be a primary cause of severe aGVHD [29, 30], we report the successful development and application of a new selection/prediction system based on HLA 3D structure modeling (HLA-TDSM) discrepancy for prediction of aGVHD severity and selection of optimal donor-recipient pairs in a prospective and retrospective randomized pilot clinical allo-HSCT study.

## RESULTS

### The establishment of HLA-TDSM system by the calculation of both coordinate and revised RMSD

**Figure 1:**
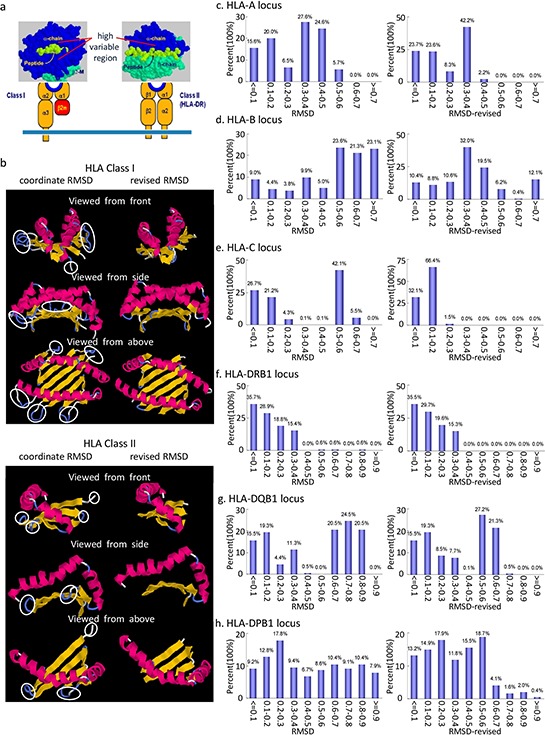
Schematic presentation of the diversity of amino acid residues (AAR) and the distribution of both coordinate RMSD and revised RMSD values at HLA class I and II molecules **a.** Allelic polymorphism at HLA-I/II molecules is concentrated in the peptide antigen binding site and TCR interaction AAR. Allelic variants may differ by 20 amino acids (Data from The Major Histocompatibility Complex of Genes by Dr. Colin R.A. Hewitt at crah1@le.ac.uk). **b.** AAR involved in coordinate RMSD (left) and revised RMSD (right) calculation at HLA class I (upper) and II molecules (bottom) with drawing by RasMol 2.7.5. AARs in white circles are excluded from revised RMSD calculation. The region of AAR used for revised RMSD calculation are aa3–13, aa20–38, aa45–85, aa92–103, aa109–127 and aa132–178 at HLA class I molecules, aa9–18, aa21–30, aa33–39, aa42–47 and aa50–87 at HLA class II molecules. At HLA class I/ II molecules, alpha helices are colored magenta, beta sheets are colored yellow, turns are colored pale blue and all other residues are colored white. **c–h.** show the distribution of both coordinate RMSD and revised RMSD values at HLA-A/-B/-Cw/-DRB1/-DQB1/-DPB1 loci respectively.

We know that not every amino acid residue (AAR) of the HLA class I and II molecules participates in antigen peptide presentation or TCR binding, especially AAR in the random coil region (Figure 1a and 1b); however, these AAR were still involved in the coordinate RMSD calculation (Figures 1 and 2 in the Supplementary Appendix). To avoid such unnecessary deviation, the calculation below was modified and AARs outside of the functional recognition region were excluded. Thus, we calculated coordinate RMSD and revised RMSD values (Figure 1b–1h), respectively, with the following formula:

$$RMSD\left(N;x,y\right)=\sqrt{\frac{\sum_{i=1}^{N}w_{i}{\left|x_{i}-y_{i}\right|}^{2}}{N\sum_{i=1}^{N}w_{i}}}$$

The extent of structure discrepancy among all HLA loci, in sequences, is DPB1 ≥ DQB1 > DRB1 ≥ Cw > B > A, according to both coordinate RMSD and revised RMSD values (Figure 1c–1h). The overall structure discrepancy of any 2 alleles from the same allele group is less than that of any 2 alleles from different allele groups, with a few exceptions. All of the revised RMSD values were changed compared to the coordinate RMSD in each locus, most prominently in HLA-Cw locus, except HLA-DRB1 locus (Figure 1c–1h). The total data of the coordinate and revised RMSD values over 6 million records were managed with Microsoft Office Access software, and results between any 2 alleles at HLA-A/-B/-Cw/-DRB1/-DQB1/-DPB1 loci can be queried easily in HLA-TDSM system, also known as HLAStrucMark [31]. With this system, more detailed information on mismatched AAR between any 2 HLA alleles can be shown, such as the mismatched AAR position, the functional characteristics of the position, and anchoring residues contributing to peptide presentation and TCR binding (Figure 1 and 2 in the Supplementary Appendix).

### The defined HLA acceptable/unacceptable mismatched alleles based on the revised RMSD

According to the distribution characteristics of both coordinate RMSD and revised RMSD values at HLA-A/-B/-Cw/-DRB1/-DQB1/-DPB1 loci, especially our combined preliminary clinical prediction results, we theoretically determined all HLA acceptable (Table 1) versus unacceptable mismatched alleles (Table 1 in the Supplementary Appendix) at each HLA-A/-B/-Cw/-DRB1/-DQB1/-DPB1 locus. The matched criteria of the revised RMSD value for acceptable mismatched alleles are ≤ 0.2Å for HLA-A*/-B*/-Cw loci and ≤ 0.1Å for HLA-DRB1*/-DQB1/-DPB1 loci. On the basis of these results, we summarized the theoretically acceptable HLA mismatched alleles with a maximum value of single/total revised RMSD < 0.5Å as the basic threshold for optimal selection of recipient-donor pair and for prediction of aGVHD severity before allo-HSCT.

**Table 1:** The defined HLA acceptable/permissible mismatching allele pairs based on revised RMSD in HLA-A*/-B*/-DRB1* loci respectively

Allele	HLA permissible mismatching allele pairs among different allele groups
	**HLA-A locus**
A*01:01	A*01:02-, A*03:01-, A*11:01-, A*24:17, A*24:19, A*24:41, A*25:01-, A*26:01-, A*29:01-, A*30:01-, A*31:01-, A*32:01-, A*33:01-, A*34:01-, A*36:01-, A*43:01-, A*66:01-, A*68:01, A*68:08-14, A*68:16, A*68:19, A*68:21, A*68:22, A*68:24-26, A*74:01-, A*80:01-
A*02:01	A*02:02-(except A*02:08), A*23:10, A*23:12, A*24:02-05, A*24:08-10, A*24:13, A*24:14, A*24:18, A*24:20, A*24:21, A*24:23, A*24:25-35, A*24:37-39, A*24:42-44, A*24:47, A*24:49, A*24:50, A*69:01
A*03:01	A*03:02-, A*11:01-, A*25:01-, A*26:01-, A*29:01-, A*30:01-, A*31:01-, A*32:01-, A*33:01-, A*34:01-, A*36:01-, A*43:01, A*66:01-, A*68:01-, A*74:01-, A*80:01-
A*11:01	A*11:02-, A*24:17, A*24:19, A*24:41, A*25:01-, A*26:01-, A*29:01-, A*30:01-, A*31:01-, A*32:01-, A*33:01-, A*34:01-, A*36:01-, A*43:01, A*66:01-, A*68:01, A*68:03-15, A*68:16-22, A*68:24-26, A*74:01-, A*80:01-
A*23:01	A*23:02-, A*24:02-05, A*24:08-15, A*24:18, A*24:20-21, A*24:23, A*24:25-39, A*24:42-44, A*24:47-50, A*69:01
A*24:02	A*24:03-05, A*24:08-15, A*24:18, A*24:20-21, A*24:23, A*24:25-39, A*24:42-44, A*24:47-50, A*69:01
A*25:01	A*25:02-, A*26:01-, A*29:01-, A*30:01-, A*31:01-, A*32:01-, A*33:01-, A*34:01-, A*36:01-, A*43:01, A*66:01-, A*68:01, A*68:03-14, A*68:16-22, A*68:24-26, A*74:01-, A*80:01-
A*26:01	A*26:02-, A*29:01-, A*30:01-, A*31:01-, A*32:01-, A*33:01-, A*34:01-, A*36:01-, A*43:01, A*66:01-, A*68:01, A*68:03-14, A*68:16-22, A*68:24-26, A*74:01-, A*80:01-
A*29:01	A*29:02-, A*30:01-, A*31:01-, A*32:01-, A*33:01-, A*34:01-, A*36:01-, A*43:01, A*66:01-, A*68:01-14, A*68:16-27, A*74:01, A*80:01-
A*30:01	A*30:02-, A*31:01-, A*32:01-, A*33:01-, A*34:01-, A*36:01-, A*43:01, A*66:01-, A*68:01, A*68:08-14, A*68:16, A*68:19, A*68:21-22, A*68:24-26, A*74:01, A*80:01-
A*31:01	A*31:02-, A*32:01-, A*33:01-, A*34:01-, A*36:01-, A*43:01, A*66:01-, A*68:01-14, A*68:16-27, A*74:01, A*80:01-
A*32:01	A*32:02-, A*33:01-, A*34:01-, A*36:01-, A*43:01, A*66:01-, A*68:01, A*68:03-05, A*68:08-14, A*68:16, A*68:19-22, A*68:24-26, A*74:01-, A*80:01-
A*33:01	A*33:02-, A*34:01-, A*36:01-, A*43:01, A*66:01-, A*68:01, A*68:01-14, A*68:16-27, A*74:01, A*80:01-
A*34:01	A*34:02-, A*36:01-, A*43:01, A*66:01-, A*68:01, A*68:08-14, A*68:16, A*68:19, A*68:21, A*68:22, A*68:24-26, A*74:01, A*80:01-
A*36:01	A*36:02-, A*43:01, A*66:01-, A*68:01, A*68:08-14, A*68:16, A*68:19, A*68:21, A*68:22, A*68:24-26, A*74:01, A*80:01-
A*43:01	A*66:01-, A*68:01, A*68:03-14, A*68:16-22, A*68:24-26, A*74:01, A*80:01-
A*66:01	A*66:02-, A*68:01, A*68:03-14, A*68:16-22, A*68:24-26, A*74:01, A*80:01-
A*68:01	A*68:02-14, A*68:16-27, A*74:01-03, A*74:05-, A*80:01-
A*74:01	A*74:02-, A*80:01-
	**HLA-B locus**
B*07:02	B*07:04, B*07:07, B*07:08, B*07:10, B*07:18, B*07:19, B*07:21-23, B*07:30, B*07:35, B*07:39, B*08:02, B*08:06, B*08:09, B*08:11, B*08:15, B*08:20, B*37:07, B*39:03, B*39:10, B*39:16, B*39:24, B*39:27, B*39:29, B*40:25, B*40:43, B*48:12, B*55:04, B*56:13, B*67:01, B*82:01, B*82:02, B*83:01
B*08:01	B*08:04, B*08:05, B*08:07, B*08:10, B*08:12-14, B*08:16-18, B*08:21-24, B*14:01-06, B*15:83, B*37:09, B*39:01, B*39:02, B*39:04, B*39:06, B*39:09, B*39:12, B*39:14, B*39:15, B*39:17, B*39:18, B*39:22, B*39:23, B*39:26, B*39:28, B*39:30, B*39:31, B*39:33, B*40:01, B*40:02, B*40:05-12, B*40:14-16, B*40:21, B*40:23, B*40:29-34, B*40:36-42, B*40:45, B*40:46, B*40:48-51, B*40:53-57, B*41:01-07, B*42:01-06, B*45:06, B*48:01, B*48:03-11, B*54:02, B*54:04, B*55:01-03, B*55:05, B*55:08-10, B*55:12, B*55:13, B*55:15-17, B*55:19, B*56:05, B*81:01, B*81:02
B*13:01	B*13:02-08, B*13:10-13, B*15:13, B*15:16, B*15:17, B*15:24, B*15:36, B*15:67, B*15:87, B*15:95, B*37:08, B*38:04, B*38:10, B*40:19, B*40:47, B*44:02-41, B*45:01-05, B*45:07, B*47:01, B*47:03, B*47:04, B*49:01-03, B*50:02, B*51:20, B*51:36, B*52:01, B*52:02, B*52:04, B*52:05, B*57:02, B*57:04, B*57:05, B*57:07, B*57:09, B*58:07, B*58:08
B*14:01	B*14:02-, B*37:05, B*37:09, B*39:01, B*39:02, B*39:04, B*39:06, B*39:09, B*39:12, B*39:14, B*39:15, B*39:17, B*39:18, B*39:22, B*39:23, B*39:26, B*39:28, B*39:30, B*39:31, B*39:33, B*40:01, B*40:02, B*40:05-12, B*40:14-16, B*40:21, B*40:23, B*40:29-34, B*40:36-42, B*40:45, B*40:46, B*40:48-51, B*40:53-57, B*41:01-07, B*42:01-, B*45:06, B*48:01, B*48:03-11, B*54:02, B*54:04, B*55:01-03, B*55:05, B*55:08-10, B*55:12, B*55:13, B*55:15-17, B*55:19, B*56:05, B*81:01, B*81:02
B*15:01	B*15:02-12, B*15:14, B*15:15, B*15:19, B*15:21, B*15:25, B*15:27, B*15:28, B*15:31-35, B*15:38-50, B*15:53-61, B*15:63-66, B*15:68-71, B*15:73-78, B*15:81, B*15:82, B*15:84-86, B*15:90-92, B*15:96-98, B*18:01, B*18:08, B*18:10, B*18:11, B*18:15, B*18:18, B*18:20, B*35:08, B*35:13, B*35:18, B*35:28, B*35:38, B*35:46, B*35:58, B*39:08, B*39:13, B*40:03, B*40:20, B*40:44, B*40:52, B*46:01-04, B*48:02, B*50:01, B*50:04, B*53:01, B*53:02, B*53:09, B*53:10, B*55:14, B*56:01, B*83:01
B*18:01	B*18:02, B*18:04, B*18:06-08, B*18:10-18, B*18:20, B*35:01-04, B*35:06-08, B*35:12-16, B*35:18-20, B*35:22, B*35:23, B*35:25, B*35:26, B*35:28, B*35:29, B*35:31-42, B*35:45-50, B*35:54-58, B*39:08, B*39:13, B*39:19, B*39:32, B*40:03, B*40:04, B*40:20, B*40:24, B*40:26, B*40:28, B*40:52, B*48:02, B*50:01, B*50:04, B*53:01-03, B*53:05, B*53:09, B*53:10, B*54:01, B*54:03, B*54:06, B*55:07, B*55:11, B*55:14, B*55:18, B*56:01-04, B*56:06, B*56:09, B*56:10, B*56:12, B*56:14, B*56:15, B*57:08, B*58:01, B*58:04, B*58:05, B*58:09, B*78:02, B*78:04, B*83:01
B*27:01	B*27:02, B*27:03, B*27:05-13, B*27:15-28, B*37:02, B*37:06, B*38:03, B*47:05, B*73:01
B*35:01	B*35:02-04, B*35:06-08, B*35:12-16, B*35:18-20, B*35:22, B*35:23, B*35:25, B*35:26, B*35:28, B*35:29, B*35:31-42, B*35:45-50, B*35:54-58, B*39:08, B*39:19, B*39:32, B*40:04, B*40:20, B*40:24, B*40:26, B*40:28, B*48:02, B*50:04, B*53:01-03, B*53:05, B*53:09, B*53:10, B*54:01, B*54:03, B*54:06, B*55:07, B*55:11, B*55:14, B*55:18, B*56:01-04, B*56:06, B*56:09, B*56:10, B*56:12-15, B*57:08, B*58:01, B*58:04, B*58:05, B*58:09, B*78:04, B*83:01,
B*37:01	B*37:04, B*37:06, B*37:08, B*38:03, B*40:13, B*40:19, B*44:06, B*47:01, B*47:03, B*47:04, B*73:01
B*38:01	B*38:02, B*38:04-10, B*49:01, B*49:03, B*51:01-37, B*52:01-06, B*53:01-03, B*53:05-10, B*56:07, B*57:01-03, B*57:05-07, B*57:09, B*58:01-09, B*59:01, B*78:02, B*78:03, B*78:05
B*39:01	B*39:02, B*39:04, B*39:06, B*39:09, B*39:12, B*39:14, B*39:15, B*39:17, B*39:22, B*39:23, B*39:26, B*39:28, B*39:30, B*39:31, B*39:33, B*40:01, B*40:02, B*40:05-12, B*40:14-16, B*40:21, B*40:23, B*40:29-34, B*40:36-42, B*40:45, B*40:46, B*40:48-51, B*40:53-57, B*41:01-07, B*42:01-06, B*45:06, B*48:01, B*48:03-11, B*54:02, B*54:04, B*55:01-03, B*55:05, B*55:08-10, B*55:12, B*55:13, B*55:15-17, B*55:19, B*56:05, B*81:01, B*81:02
B*40:01	B*40:02, B*40:05-12, B*40:14-16, B*40:21, B*40:23, B*40:29-34, B*40:36-42, B*40:45, B*40:46, B*40:48-51, B*40:53-57, B*41:01-07, B*42:01-06, B*45:06, B*48:01, B*48:03-11, B*54:02, B*54:04, B*55:01-03, B*55:05, B*55:08-10, B*55:12, B*55:13, B*55:15-17, B*55:19, B*56:05, B*81:01, B*81:02
B*41:01	B*41:02-07, B*42:01-06, B*45:06, B*48:01, B*48:03-11, B*54:02, B*54:04, B*55:01-03, B*55:05, B*55:08-10, B*55:12, B*55:13, B*55:15-17, B*55:19, B*56:05, B*81:01, B*81:02
B*42:01	42:02-06, B*45:06, B*48:01, B*48:03-11, B*54:02, B*54:04, B*55:01-03, B*55:05, B*55:08-10, B*55:12, B*55:13, B*55:15-17, B*55:19, B*56:05, B*81:01, B*81:02
B*44:02	B*44:03-41, B*45:01-05, B*45:07, B*47:01, B*47:03, B*47:04, B*49:01, B*49:02, B*50:02, B*52:02, B*57:02, B*57:04, B*57:05, B*57:07, B*58:07
B*45:01	B*45:02-05, B*45:07, B*47:02-04, B*49:01, B*49:02, B*50:02, B*57:04,
B*46:01	B*46:02-04, B*50:01, B*50:04
B*47:01	B*47:03-05, B*49:02, B*57:04, B*73:01
B*48:01	B*48:03-11, B*54:02, B*54:04, B*55:01-03, B*55:05, B*55:08-10, B*55:12, B*55:13, B*55:15-17, B*55:19, B*56:05, B*81:01, B*81:02
B*49:01	B*49:02, B*49:03, B*51:03, B*51:20, B*51:23, B*51:31, B*51:36, B*52:01, B*52:02, B*52:04, B*52:05, B*57:02, B*57:04, B*57:05, B*57:07, B*57:09, B*58:07, B*58:08, B*59:01
B*50:01	B*50:04, B*53:01, B*53:02, B*53:09, B*53:10, B*55:14, B*56:01, B*83:01
B*51:01	B*51:02-37, B*52:01-06, B*53:01-03, B*53:05-10, B*56:06, B*56:07, B*57:01-03, B*57:05-09, B*58:01-09, B*59:01, B*78:01-03, B*78:05
B*52:01	B*52:02-06, B*53:06, B*53:07, B*56:07, B*57:01-07, B*57:09, B*58:02, B*58:06-08, B*59:01, B*78:05
B*53:01	B*53:02, B*53:03, B*53:05-10, B*54:01, B*54:03, B*54:06, B*55:07, B*55:14, B*55:18, B*56:01-04, B*56:06, B*56:07, B*56:09, B*56:10, B*56:13-15, B*57:01, B*57:03, B*57:06, B*57:08, B*58:01-06, B*58:09, B*59:01, B*78:01-05, B*83:01
B*54:01	B*54:03, B*54:06, B*55:07, B*55:11, B*55:14, B*55:18, B*56:01-04, B*56:06, B*56:09, B*56:10, B*56:12, B*56:14, B*56:15, B*57:08, B*58:01, B*58:04, B*58:05, B*58:09, B*78:01-04, B*83:01
B*55:01	B*55:02, B*55:03, B*55:05, B*55:08-10, B*55:12, B*55:13, B*55:15-17, B*55:19, B*56:05, B*81:01, B*81:02
B*56:01	B*56:02-04, B*56:06, B*56:09, B*56:10, B*56:12, B*56:14, B*56:15, B*57:08, B*58:01, B*58:04, B*58:05, B*58:09, B*78:02, B*78:04, B*83:01
B*57:01	B*57:02, B*57:03, B*57:05-09, B*58:01-09, B*59:01, B*78:01-05
B*58:01	B*58:02-06, B*58:09, B*59:01, B*78:01-05, B*83:01
B*59:01	B*78:03, B*78:05
B*67:01	B*82:01, B*82:02
B*73:01	nothing
B*78:01	B*78:02-04
B*81:01	B*81:02
B*82:01	B*82:02
B*83:01	nothing
	**HLA-DRB1 locus**
DRB1*01:01	DRB1*01:03-05, DRB1*01:07, DRB1*01:12, DRB1*07:06, DRB1*10:01
DRB1*03:01	DRB1*03:02, DRB1*03:03, DRB1*03:05-18, DRB1*03:20-28, DRB1*11:17, DRB1*11:20, DRB1*11:25, DRB1*11:46, DRB1*11:52, DRB1*11:54, DRB1*13:02, DRB1*13:08, DRB1*13:16, DRB1*13:19, DRB1*13:29, DRB1*13:31, DRB1*13:34, DRB1*13:36, DRB1*13:39-41, DRB1*13:52, DRB1*13:53, DRB1*13:56, DRB1*13:57, DRB1*13:63-65, DRB1*14:01-06, DRB1*14:08, DRB1*14:09, DRB1*14:11, DRB1*14:13, DRB1*14:15-21, DRB1*14:23, DRB1*14:24, DRB1*14:26, DRB1*14:28-35, DRB1*14:37-39, DRB1*14:43, DRB1*14:45, DRB1*14:47, DRB1*14:48
DRB1*04:01	DRB1*04:02-19, DRB1*04:21-53, DRB1*08:01-15, DRB1*08:17-30, DRB1*11:01-08, DRB1*11:10-14, DRB1*11:18, DRB1*11:19, DRB1*11:21-23, DRB1*11:26, DRB1*11:27, DRB1*11:29-39, DRB1*11:41-45, DRB1*11:48-51, DRB1*11:53, DRB1*13:03, DRB1*13:04, DRB1*13:07, DRB1*13:11-14, DRB1*13:17, DRB1*13:21-25, DRB1*13:30, DRB1*13:32, DRB1*13:33, DRB1*13:37, DRB1*13:38, DRB1*13:44-49, DRB1*13:54, DRB1*13:55, DRB1*13:58, DRB1*13:60, DRB1*13:66, DRB1*14:07, DRB1*14:10, DRB1*14:14, DRB1*14:22, DRB1*14:25, DRB1*14:36, DRB1*14:40-42, DRB1*14:44, DRB1*14:46
DRB1*07:01	DRB1*07:03, DRB1*07:05, DRB1*07:06, DRB1*07:09, DRB1*10:01
DRB1*08:01	DRB1*08:02-15, DRB1*08:17-30, DRB1*11:01-08, DRB1*11:10-14, DRB1*11:18, DRB1*11:19, DRB1*11:21-23, DRB1*11:26, DRB1*11:27, DRB1*11:29-39, DRB1*11:41-45, DRB1*11:48-51, DRB1*11:53, DRB1*13:03, DRB1*13:04, DRB1*13:07, DRB1*13:11-14, DRB1*13:17, DRB1*13:21-25, DRB1*13:30, DRB1*13:32, DRB1*13:33, DRB1*13:37, DRB1*13:38, DRB1*13:44-49, DRB1*13:54, DRB1*13:55, DRB1*13:58, DRB1*13:60, DRB1*13:66, DRB1*14:07, DRB1*14:10, DRB1*14:14, DRB1*14:22, DRB1*14:25, DRB1*14:36, DRB1*14:40-42, DRB1*14:44, DRB1*14:46
DRB1*09:01	DRB1*09:02-04, DRB1*11:15, DRB1*13:62
DRB1*10:01	nothing
DRB1*11:01	DRB1*11:02-08, DRB1*11:10-14, DRB1*11:18, DRB1*11:19, DRB1*11:21-23, DRB1*11:25-27, DRB1*11:29-39, DRB1*11:41-46, DRB1*11:48-51, DRB1*11:53, DRB1*13:03, DRB1*13:04, DRB1*13:07, DRB1*13:08, DRB1*13:11-14, DRB1*13:17, DRB1*13:19, DRB1*13:21-25, DRB1*13:30, DRB1*13:32, DRB1*13:33, DRB1*13:37, DRB1*13:38, DRB1*13:44-49, DRB1*13:52-55, DRB1*13:58, DRB1*13:60, DRB1*13:63, DRB1*13:66, DRB1*14:02, DRB1*14:07, DRB1*14:09, DRB1*14:10, DRB1*14:13-15, DRB1*14:22, DRB1*14:25, DRB1*14:28, DRB1*14:32, DRB1*14:36, DRB1*14:37, DRB1*14:40-42, DRB1*14:44, DRB1*14:46
DRB1*12:01	DRB1*12:03-12, DRB1*14:27
DRB1*13:01	DRB1*13:05, DRB1*13:06, DRB1*13:09, DRB1*13:10, DRB1*13:15, DRB1*13:18, DRB1*13:20, DRB1*13:27, DRB1*13:28, DRB1*13:35, DRB1*13:42, DRB1*13:51, DRB1*13:59
DRB1*14:01	DRB1*14:02-09, DRB1*14:11, DRB1*14:13, DRB1*14:15, DRB1*14:16, DRB1*14:18-21, DRB1*14:23, DRB1*14:24, DRB1*14:26, DRB1*14:28-35, DRB1*14:37-40, DRB1*14:42, DRB1*14:43, DRB1*14:45, DRB1*14:47, DRB1*14:48
DRB1*15:01	DRB1*15:02, DRB1*16:01-03
DRB1*16:01	DRB1*16:02, DRB1*16:03

HLA-A*/-B*/-DRB1* loci include at least 2132, 2798 and 1196 alleles respectively, all of whom are the most polymorphic in all HLA loci. For these reasons, we selected HLA-A*/-B*/-DRB1* loci as the representative of acceptable/permissible mismatching allele pairs from all HLA loci. The matched criteria of the revised RMSD value are ≤ 0.2Å for HLA-A*/-B*loci and ≤ 0.1Å for HLA-DRB1*locus. The limited layout sake this table shows only partial results. The dash “-“indicates all the other alleles in this allele group.

### Evaluation of accuracy and reliability of HLA-TDSM system by antigen-specific alloreactive CTLs

FACS analysis and TCR Vβ gene scan showed that HLA-B molecules pulsed with Epstein-Barr virus nonameric peptide (EBNA3) could induce clonal CTL activation in stable transfected Hmy2.CIR cells expressing HLA-B*15:02, -B*15:18, -B*35:03, or -B*44:03 (data not shown), and exhibited various cross-reaction characteristics and HLA restriction (Tables 2–4 and Figures 3–4 in the Supplementary Appendix). The revised RMSD between B*44:03 and HLA-B*15:02,-B*15:18, or -B*35:03 were much higher than those within HLA-B*15:02, -B*15:18, and -B*35:03 in which there are many AAR discrepancies in the position of F epitope-binding pocket. These results confirmed that cross-reaction patterns by antigen-specific alloreactive CTLs could be used efficiently to evaluate the accuracy and reliability of the HLA-TDSM system. With these available data, we further verified the accuracy and reliability of the HLA-TDSM system.

### A close correlation between the revised RMSD and aGVHD severity in retrospectively randomized allo-HSCT

In the retrospective study, we randomly collected and analyzed the correlation between the revised RMSD and aGVHD severity in 12 transplanted recipient-donor pairs. As shown in Tables 2 and 3, of 12 recipient-donor pairs, there were 6 recipient-donor pairs who had 1 allele mismatching at HLA-A, -B, -DRB1, or -DQB1 locus, 3 recipient-donor pairs who had 2 alleles mismatching at HLA-A+DRB1, -B+DQB1, or -DRB1+DQB1 loci; and 3 recipient-donor pairs who had 3 alleles mismatching at either HLA-A+B+DRB1 or HLA-B+DRB1+DQB1 loci. Our results indicate that the occurrence of severe aGVHD in recipient-donor pairs with either 1 or 2 alleles mismatching at any locus had a close correlation with single or total revised RMSD > 0.50Å, but individual HLA-allele, allele group, loci, or the combination of different allele/loci did not. Severe aGVHD did not occur even if recipient-donor pair had 3 alleles mismatching, as long as total revised RMSD < 0.50Å. In contrast, severe aGVHD still occurred even if recipient-donor pair had HLA-allele mismatching at the same allele group as long as single or total revised RMSD > 0.50Å. The 1 exception is R-UPN07 recipient-donor pair, who had only 1 allele mismatching between HLA-A*02:01 and -A*68:01 and single revised RMSD 0.43Å < 0.50Å, but still developed grade IV severe aGVHD. We considered that R-UPN07 was a special case because she was confirmed to have 1 HLA-A/B loci recombination, which produces a novel haplotype that may have caused the severe aGVHD [31]. Several representative images comparing the results of 3D structure modeling for 1 allele mismatching at HLA-A/-B/-DRB1/-DQB1 loci from either retrospective or prospective recipient-donor pairs are shown using RASMOL software (Figure 5 in the Supplementary Appendix).

**Table 2:** Demographic characteristics and disease status of patient-donor pairs in retrospective and prospective groups

UPN+	Patients				Donors	
	Age/Sex	Disease status	Transplant status	HLA-A/B/DRB1/DQB1 low-resolution	Donor/Age	HLA-A/B/DRB1/DQB1 low-resolution
R-UPN01	12/male	ALL/RE3	Yes	A*02/11, B*13/60, DRB1*12/15, DQB1*06/07	Father/40	A*02/33, B*44/60, DRB1*12/13, DQB1*06/07
R-UPN02	10/male	ALL/CR2	Yes	A*11/24, B*60/62, DRB1*11/15, DQB1*05/07	Mother/32	A*11/11, B*46/62, DRB1*12/15, DQB1*05/07
R-UPN03	15/male	AML/CR1	Yes	A*02/11, B*60/60, DRB1*09/11, DQB1*07/09	Sister/11	A*11/11, B*46/60, DRB1*09/11, DQB1*07/09
R-UPN04	26/female	AML/CR1	Yes	A*01/02, B*13/37, DRB1*10/16, DQB1*05/05	Brother/35	A*01/33, B*13/37, DRB1*10/16, DQB1*05/05
R-UPN05	36/male	AML/CR1	Yes	A*11/30, B*13/62, DRB1*13/13, DQB1*06/06	Sister/37	A*11/30, B*13/44, DRB1*04/13, DQB1*06/08
R-UPN06	21/female	AML/RE1	Yes	A*02/33, B*07/60, DRB1*04/04, DQB1*02/07	Unrelated BM	A*02/33, B*07/60, DRB1*04/04, DQB1*02/07
R-UPN07	30/female	AML/CR1	Yes	A*02/203, B*38/52, DRB1*04/07, DQB1*02/04	Brother/38	A*203/68, B*38/52, DRB1*04/07, DQB1*02/04
R-UPN08	45/female	AML/CR1	Yes	A*02/30, B*38/60, DRB1*11/15, DQB1*06/07	Brother/38	A*02/30, B*39/60, DRB1*11/15, DQB1*06/07
R-UPN09	22/male	AML/CR1	Yes	A*02/24, B*35/61, DRB1*08/15, DQB1*06/06	Father/52	A*02/02, B*35/61, DRB1*15/16, DQB1*05/06
R-UPN10	42/male	CML/CP	Yes	A*02/11, B*07/62, DRB1*01/15, DQB1*05/08	Brother/30	A*02/11, B*07/62, DRB1*01/15, DQB1*05/06
R-UPN11	34/male	CML/CP	Yes	A*11/33, B*58/61, DRB1*12/16, DQB1*05/07	Brother/27	A*11/33, B*13/58, DRB1*12/16, DQB1*05/07
R-UPN12	36/male	NHL/CR1	Yes	A*02/30, B*13/55, DRB1*07/12, DQB1*02/07	Brother/42	A*02/30, B*13/55, DRB1*07/11, DQB1*02/08
P-UPN01	17/male	ALL/CR2	Yes	A*11/31, B*46/48, DRB1*08/14, DQB1*05/06	Father/43	A*02/11, B*46/51, DRB1*08/12, DQB1*06/07
P-UPN02	32/male	ALL/CR1	Yes	A*24/33, B*35/55, DRB1*04/12, DQB1*02/05	Brother/30	A*02/33, B*35/55, DRB1*04/12, DQB1*02/05
P-UPN03	9/male	ALL/CR1	Yes	A*24/33, B*44/62, DRB1*11/14, DQB1*06/07	Unrelated CB	A*11/24, B*44/62, DRB1*04/11, DQB1*06/07
P-UPN04	24/female	ALL/CR1	Yes	A*02/33, B*51/60, DRB1*04/15, DQB1*06/08	Brother/23	A*02/03, B*35/60, DRB1*04/15, DQB1*06/08
P-UPN05	24/female	AML/CR1	Yes	A*02/31, B*46/51, DRB1*12/15, DQB1*05/07	Father/50	A*02/31, B*38/51, DRB1*12/16, DQB1*05/07
P-UPN06	39/female	AML/CR1	Yes	A*11/11, B*18/60, DRB1*09/14, DQB1*04/08	Sister/37	A*11/24, B*35/60, DRB1*04/09, DQB1*04/08
P-UPN07	18/male	AML/CR1	Yes	A*02/02, B*13/61, DRB1*08/12, DQB1*05/07	Unrelated CB	A*02/203, B*13/60, DRB1*08/12, DQB1*05/07
P-UPN08	52/female	AML/CR2	No	A*11/30, B*13/38, DRB1*07/12, DQB1*02/07	Brother/47	A*11/30, B*13/27, DRB1*07/12, DQB1*02/07
P-UPN09	20/female	AML/CR1	No	A*203/11, B*38/60, DRB1*04/15, DQB1*06/07	Unrelated CB	A*11/24, B*38/60, DRB1*04/15, DQB1*06/07
P-UPN10	45/female	AML/CR1	Yes	A*02/24, B*27/44, DRB1*01/11, DQB1*05/07	Brother/32	A*02/02, B*44/44, DRB1*01/11, DQB1*05/07
P-UPN11	29/male	CML/CP	Yes	A*02/11, B*13/62, DRB1*11/11, DQB1*07/07	Father/55	A*11/30, B*13/13, DRB1*11/11, DQB1*07/07
P-UPN12	33/female	CML/CP	Yes	A*11/24, B*51/60, DRB1*04/15, DQB1*06/08	Brother/35	A*02/24, B*51/60, DRB1*04/15, DQB1*06/08
P-UPN13	35/female	CML/CP	Yes	A*02/26, B*51/62, DRB1*09/13, DQB1*06/09	Sister/30	A*02/30, B*13/62, DRB1*07/09, DQB1*02/09
P-UPN14	37/male	CML/CP	Yes	A*01/11, B*37/54, DRB1*08/10, DQB1*05/06	Brother/35	A*01/26, B*37/54, DRB1*08/10, DQB1*05/06
P-UPN15	28/male	CML/CP	Yes	A*11/24, B*37/60, DRB1*04/10, DQB1*02/05	Sister/30	A*11/24, B*46/60, DRB1*04/09, DQB1*02/05
P-UPN16	25/male	CML/CP	Yes	A*02/02, B*54/60, DRB1*08/11, DQB1*06/07	Sister/22	A*02/02, B*46/60, DRB1*08/11, DQB1*06/07
P-UPN17	40/male	CML/CP	Yes	A*11/31, B*35/61, DRB1*11/13, DQB1*07/07	Son/14	A*02/31, B*13/61, DRB1*11/13, DQB1*06/07
P-UPN18	30/male	CML/CP	Yes	A*11/11, B*35/71, DRB1*15/17, DQB1*02/06	Brother/39	A*01/11, B*35/57, DRB1*07/15, DQB1*02/06
P-UPN19	28/male	CML/CP	Yes	A*02/02, B*13/46, DRB1*04/09, DQB1*08/09	Brother/24	A*02/02, B*13/46, DRB1*04/15, DQB1*05/08
P-UPN20	24/male	CML/CP	Yes	A*30/31, B*35/61, DRB1*09/12, DQB1*07/09	Mother/47	A*24/30, B*35/51, DRB1*09/12, DQB1*07/09
P-UPN21	14/male	CML/CP	Yes	A*02/24, B*39/46, DRB1*09/12, DQB1*07/09	Mother/38	A*02/02, B*39/46, DRB1*09/12, DQB1*07/09
P-UPN22	13/female	MDS-	Yes	A*02/02, B*07/27, DRB1*15/17, DQB1*02/06	Father/35	A*02/30, B*13/27, DRB1*07/17, DQB1*02/06
P-UPN23	15/male	Thal	No	A*02/11, B*54/60, DRB1*04/04, DQB1*04/07	Father/40	A*02/11, B*54/60, DRB1*04/14, DQB1*05/07
P-UPN24	03/male	Thal	No	A*11/11, B*13/54, DRB1*08/14, DQB1*05/06	Mother/38	A*11/11, B*54/75, DRB1*08/14, DQB1*05/06
P-UPN25	5/male	Thal	No	A*11/11, B*51/75, DRB1*11/12, DQB1*06/07	Sister CB	A*11/11, B*51/55, DRB1*11/14, DQB1*06/07

R-UPN, retrospective unique patient number; P-UPN, prospective unique patient number; ALL, acute lymphoblastic leukemia; AML, acute myelocytic leukemia; CML, chronic myelocytic leukemia; MDS-RAEB-t, myelodysplastic syndrome-refractory anemia with excess blasts in transformation; NHL, Non-Hodgkin lymphoma; Thal, thalassemia; CR, complete remission; RE, relapse; CP, chronic phase; CB, cord blood.

**Table 3:** The correlated results of among HLA-A/-B/-DRB1/-DQB1 high-resolution, HLA 3D structure discrepancy and aGVHD severity in 32 patient-donor pairs

UPN+	HLA alleles		Mismatches (MM)		MM amino acids in key sites			RMSD (Å)	Revised RMSD (Å)	Total revised RMSD (Å)	aGVHD
	Patients	Donors	MM loci	No. of MM amino acids	pocket	Peptide binding	TCR contact				
R- UPN01	A*02: 01/11:01	A*02: 01/33:01	A*11: 01/33:01	15	A,B,C,D,E	yes	yes	0.3615	0.0930	0.2958	I^ö^
	B*13: 01/40:01	B*40: 01/44:02	B*13: 01/44:02	11	A,B,D,E,F	yes	yes	0.0976	0.0836		
	DRB1* 12:01/15:01	DRB1* 12:01/13:01	DRB1* 15:01/13:01	7	A,B,C,D,E	yes	yes	0.1258	0.1192		
	DQB1* 03:01/06:01	DQB1* 03:01/06:01									
R- UPN02	A*11: 01/24:02	A*11: 01/11:01	A*24: 02/11:01	24	A,B,C,D,E,F	yes	yes	0.4273	0.3774	0.9975	III^ö^
	B*15: 01/40:01	B*15: 01/46:01	B*40: 01/46:01	23	A,B,C,D,E,F,	yes	yes	0.5812	0.4260		
	DRB1* 11:01/15:01	DRB1* 12:01/15:01	DRB1* 11:01/12:01	14	A,B,C,D,E	yes	no	0.1991	0.1941		
	DQB1* 03:01/05:01	DQB1* 03:01/05:01									
R- UPN03	A*02: 01/11:01	A*11: 01/11:01	A*02: 01/11:01	18	A,B,C,D,E,F	yes	yes	0.4155	0.3755	0.3755	II^ö^
	B*40: 01/40:01	B*40: 01/46:01	no MM in GVH direction								
	DRB1* 09:01/11:01	DRB1* 09:01/11:01									
	DQB1* 03:01/03:03	DQB1* 03:01/03:03									
R- UPN04	A*01: 01/02:01	A*01: 01/33:01	A*02: 01/33:01	17	A,B,C,D,E,F	yes	yes	0.5467	0.3807	0.3807	II^ö^
	B*13: 01/37:01	B*13: 01/37:01									
	DRB1* 10:01/16:01	DRB1* 10:01/16:01									
	DQB1* 05:01/05:01	DQB1* 05:01/05:01									
R- UPN05	A*11: 01/30:01	A*11: 01/30:01								0.368	II^ö^
	B*13: 01/15:01	B*13: 01/44:02	B*15: 01/44:02	17	A,B,D,E,F	yes	yes	0.3769	0.3680		
	DRB1*13: 01/13:01	DRB1*04: 06/13:01	no MM in GVH direction								
	DQB1*06: 01/06:01	DQB1*03: 02/06:01	no MM in GVH direction								
R- UPN06	A*02: 01/33:01	A*02: 01/33:01								0.0838	II^ö^
	B*07: 02/40:01	B*07: 02/40:01									
	DRB1*04: 01/04:01	DRB1*04: 04/04:04	DRB1*04: 01/04:04	2	A,B,D	yes	no	0.0880	0.0838		
	DQB1*02: 01/03:01	DQB1*02: 01/03:01									
R- UPN07	A*02: 01/02:03	A*02: 03/68:01	A*02: 01/68:01	12	A,B,C,D,E,F	yes	yes	0.4734	0.4317	0.4317	IV^ö^
	B*38: 01/52:01	B*38: 01/52:01									
	DRB1*04: 01/07:01	DRB1*04: 01/07:01									
	DQB1*02: 01/04:01	DQB1*02: 01/04:01									
R- UPN08	A*02: 01/30:01	A*02: 01/30:01								0.6474	IV^ö^
	B*38: 01/40:01	B*39: 02/40:01	B*38: 01/39:02	8	A,B,C,F	yes	no	0.5809	0.3517		
	DRB1* 11:01/15:01	DRB1* 11:01/15:01									
	DQB1* 03:01/06:01	DQB1* 03:01/06:04	DQB1* 06:01/06:04	11	A,B,C,D,E	yes	yes	0.3044	0.2957		
R-UPN09	A*02: 01/24:02	A*02: 01/02:01	A*24: 02/02:01	20	A,B,C,D,E,F	yes	yes	0.1666	0.1472	0.3750	II^ö^
	B*35: 01/40:02	B*35: 01/40:02									
	DRB1* 08:01/15:01	DRB1* 15:01/16:01	DRB1* 08:01/16:01	9	B,C,E	yes	No	0.2283	0.2278		
	DQB1* 06:01/06:01	DQB1* 05:01/06:01	no MM in GVHD direction								
R- UPN10	A*02: 01/11:01	A*02: 01/11:01								0.6333	IV^ö^
	B*07: 02/15:01	B*07: 02/15:01									
	DRB1* 01:01/15:01	DRB1* 01:01/15:01									
	DQB1* 03:11/05:01	DQB1* 05:01/06:11	DQB1* 03:11/06:11	10	A,B,E	yes	yes	0.7620	0.6333		
R- UPN11	A*11: 01/33:01	A*11: 01/33:01								0.5059	IV^ö^
	B*40: 02/58:01	B*13: 01/58:01	B*40: 02/13:01	18	B,C,F	yes	no	0.5681	0.3628		
	DRB1* 12:01/16:01	DRB1* 12:01/16:05	DRB1* 16:01/16:05	1	D	yes	no	0.1378	0.1366		
	DQB1*03:01/05:01	DQB1* 03:04/05:01	DQB1* 03:01/03:04	1	E	yes	no	0.0090	0.0065		
R- UPN12	A*02: 01/30:01	A*02: 01/30:01								0.2211	I^ö^
	B*13: 01/55:01	B*13: 01/55:01									
	DRB1* 07:01/12:01	DRB1* 07:01/11:01	DRB1* 12:01/11:01	14	A,B,C,D,E	yes	no	0.1991	0.1941		
	DQB1* 02:01/03:01	DQB1* 02:01/03:02	DQB1* 03:01/03:02	4	B,C,E	yes	no	0.0286	0.0270		
P-UPN01	A*11: 01/31:01	A*02: 07/11:01	A*31: 01/02:07	17	A,B,C,D,E,F	yes	yes	0.4137	0.3625	1.4094	IV^ö^
	B*46: 01/48:01	B*46: 01/51:01	B*48: 01/51:01	21	A,B,C,E,F	yes	yes	0.5876	0.3657		
	DRB1* 08:01/14:01	DRB1* 08:01/12:01	DRB1* 14:01/12:01	14	A,B,C,D,E	yes	yes	0.1545	0.1508		
	DQB1* 05:01/06:01	DQB1* 03:01/06:01	DQB1* 05:01/03:01	19	A,B,C,D,E	yes	yes	0.6991	0.5304		
P-UPN02	A*24: 02/33:01	A*02: 07/33:01	A*24: 02/02:07	20	A,B,C,D,E,F	yes	yes	0.1640	0.1363	0.1363	II^ö^
	B*35: 01/55:01	B*35: 01/55:01									
	DRB1* 04:01/12:01	DRB1* 04:01/12:01									
	DQB1* 02:01/05:01	DQB1* 02:01/05:01									
P-UPN03	A*24: 02/33:01	A*11: 01/24:02	A*33: 01/11:01	15	A,B,C,D,E	yes	yes	0.3615	0.0929	0.2579	I^ö^
	B*15: 01/44:03	B*15: 01/44:02	B*44: 03/44:02	1	D,E	yes	no	0.0730	0.0502		
	DRB1* 11:01/14:01	DRB1* 04:01/11:01	DRB1* 14:01/04:01	13	A,B,C,D,E	yes	yes	0.1186	0.1148		
	DQB1* 03:01/06:01	DQB1* 03:01/06:01									
P-UPN04	A*02: 01/33:01	A*02: 01/03:01	A*33: 01/03:01	12	A,B,C,D,E	yes	yes	0.3619	0.0899	0.3976	II^ö^
	B*40: 01/51:01	B*35: 01/40:01	B*51: 01/35:01	13	A,C,D,E,F	yes	no	0.6636	0.3677		
	DRB1* 04:01/15:01	DRB1*04: 01/15:01									
	DQB1* 03:02/06:01	DQB1* 03:02/06:01									
P-UPN05	A*02: 01/31:01	A*02: 01/31:01								0.3976	II^ö^
	B*46: 01/51:01	B*38: 02/51:01	B*46: 01/38:02	24	A,B,C,D,E,F	yes	yes	0.4214	0.3567		
	DRB1* 12:01/15:02	DRB1* 12:01/16:02	DRB1* 15:02/16:02	4	B,D	yes	yes	0.0345	0.0241		
	DQB1* 03:01/05:01	DQB1* 03:01/05:01									
P-UPN06	A*11: 01/11:01	A*11: 01/24:02	no MM in GVHD direction							0.1832	I^ö^
	B*18: 01/40:01	B*35: 01/40:01	B*18: 01/35:01	11	A,B,C	yes	yes	0.0864	0.0684		
	DRB1* 09:01/14:01	DRB1* 04:01/09:01	DRB1* 14:01/04:01	13	A,B,C,D,E	yes	yes	0.1186	0.1148		
	DQB1*03:02/04:01	DQB1* 03:02/04:01									
P-UPN07	A*02: 01/02:01	A*02: 01/02:03	no MM in GVHD direction							0.1102	I^ö^
	B*13: 01/40:06	B*13: 01/40:01	B*40: 06/40:01	8	C,E,F	yes	no	0.1325	0.1102		
	DRB1* 08:03/12:02	DRB1* 08:03/12:02									
	DQB1* 03:01/05:01	DQB1* 03:01/05:01									
P-UPN10	A*02: 01/24:02	A*02: 01/02:05	A*24: 02/02:05	20	A,B,C,D,E,F	yes	yes	0.1646	0.1370	0.4472	II^ö^
	B*27: 07/44:02	B*44: 02/44:02	B*27: 07/44:02	22	A,B,C,D,E,F	yes	yes	0.3292	0.3102		
	DRB1* 01:01/11:01	DRB1* 01:01/11:01									
	DQB1* 03:01/05:01	DQB1* 03:01/05:01									
P-UPN11	A*02: 01/11:01	A*11: 01/30:01	A*02: 01/30:01	18	A,B,C,D,E,F	yes	yes	0.4225	0.3820	0.7544	IV^ö^
	B*13: 01/15:01	B*13: 01/13:02	B*15: 01/13:02	16	A,B,C,D,E,F	yes	yes	0.3914	0.3724		
	DRB1* 11:01/11:01	DRB1* 11:01/11:01									
	DQB1* 03:01/03:01	DQB1* 03:01/03:01									
P-UPN12	A*11: 01/24:02	A*02: 01/24:02	A*11: 01/02:01	18	A,B,C,D,E,F	yes	yes	0.4155	0.3755	0.3755	II^ö^
	B*40: 01/51:01	B*40: 01/51:01									
	DRB1* 04:04/15:01	DRB1* 04:04/15:01									
	DQB1* 03:02/06:01	DQB1* 03:02/06:01									
P-UPN13	A*02: 01/26:01	A*02: 01/30:01	A*26: 01/30:01	18	A,B,C,D,E,F	yes	yes	0.0850	0.0775	1.2944	IV^ö^
	B*15: 01/51:01	B*13: 01/15:01	B*51: 01/13:01	16	A,B,C,E,F	yes	yes	0.2842	0.2261		
	DRB1* 09:01/13:01	DRB1* 07:01/09:01	DRB1* 13:01/07:01	19	A,B,C,D,E	yes	no	0.3435	0.3129		
	DQB1* 03:03/06:01	DQB1* 02:01/03:03	DQB1* 06:01/02:01	21	A,B,C,D,E	yes	yes	0.8049	0.6779		
P-UPN14	A*01: 01/11:01	A*01: 01/26:01	A*11: 01/26:01	11	A,B,C,D,E,F	yes	yes	0.0412	0.0403	0.0403	I^ö^
	B*37: 01/54:01	B*37: 01/54:01									
	DRB1* 08:01/10:01	DRB1* 08:01/10:01									
	DQB1* 05:01/06:01	DQB1* 05:01/06:01									
P-UPN15	A*11: 01/24:02	A*11: 01/24:02								0.8127	III^ö^
	B*37: 01/40:01	B*40: 01/46:01	B*37: 01/46:01	25	A,B,C,D,E,F	yes	yes	0.4856	0.4726		
	DRB1* 04:01/10:01	DRB1* 04:01/09:01	DRB1* 10:01/09:01	16	B,C,D,E	yes	no	0.3691	0.3401		
	DQB1* 02:01/05:01	DQB1* 02:01/05:01									
P-UPN16	A*02: 01/02:01	A*02: 01/02:01								0.2595	I^ö^
	B*40:01/54:01	B*40: 01/46:01	B*54:01/46:01	15	A,B,C,D,E,F	yes	yes	0.6756	0.2595		
	DRB1*08:01/11:01	DRB1* 08:01/11:01									
	DQB1*03:01/06:01	DQB1* 03:01/06:01									
P-UPN17	A*11:01/31:01	A*02: 01/31:01	A*11:01/02:01	18	A,B,C,D,E,F	yes	yes	0.4155	0.3755	0.7682	III^ö^
	B*35:01/40:02	B*13: 01/40:02	B*35:01/13:01	15	A,B,D,E,F	yes	yes	0.7462	0.3927		
	DRB1*11:01/13:01	DRB1* 11:01/13:01									
	DQB1*03:01/03:01	DQB1* 03:01/06:01	no MM in GVHD direction								
P-UPN18	A*11:01/11:03	A*01: 01/11:01	A*11:03/01:01	13	A,B,C,D,E,F	yes	yes	0.0171	0.0143	0.5978	IV^ö^
	B*35: 01/15:18	B*35: 01/57:01	B*15: 18/57:01	20	A,B,C,E,F	yes	yes	0.4572	0.2467		
	DRB1* 03:01/15:01	DRB1* 07:01/15:01	DRB1* 03:01/07:01	22	A,B,C,D,E	yes	yes	0.3688	0.3368		
	DQB1* 02:01/06:01	DQB1* 02:01/06:01									
P-UPN19	A*02: 01/02:01	A*02:01/02:01								0.8322	IV^ö^
	B*13: 01/46:01	B*13:01/46:01									
	DRB1* 04:01/09:01	DRB1* 04:01/15:01	DRB1* 09:01/15:01	17	A,B,C,D,E	yes	yes	0.3889	0.3024		
	DQB1* 03:02/03:03	DQB1* 03:02/05:01	DQB1* 03:02/05:01	17	A,B,D,E	yes	yes	0.6986	0.5298		
P-UPN20	A*30: 01/31:01	A*24: 02/30:01	A* 31: 01/24:02	23	A,B,C,D,E,F	yes	yes	0.4511	0.3906	0.7627	IV^ö^
	B*35: 01/40:06	B*35: 01/51:01	B* 40: 06/51:01	18	A,B,C,E,F	yes	yes	0.5993	0.3721		
	DRB1* 09:01/12:01	DRB1* 09:01/12:01									
	DQB1* 03:01/03:03	DQB1* 03:01/03:03									
P-UPN21	A*02: 01/24:02	A*02: 01/02:01	A* 24: 02/02:01	20	A,B,C,D,E,F	yes	yes	0.1666	0.1472	0.1472	II^ö^
	B*39: 01/46:01	B*39:01/46:01									
	DRB1* 09:01/12:01	DRB1* 09:01/12:01									
	DQB1* 03:01/03:03	DQB1* 03:01/03:03									
P-UPN22	A*02: 01/02:01	A*02:01/30:01	no MM in GVHD direction							0.6499	III^ö^
	B*07: 05/27:07	B*13:01/27:07	B* 07: 05/13:01	28	A,B,C,D,E,F	yes	yes	0.5769	0.3870		
	DRB1* 03:01/15:01	DRB1* 03:01/07:01	DRB1* 15:01/07:01	16	A,B,C,D,E	yes	yes	0.3062	0.2629		
	DQB1* 02:01/06:01	DQB1* 02:01/06:01									

R-UPN, retrospective unique patient number; P-UPN, prospective unique patient number; MM, mismatches; RMSD, coordinate root-mean-square deviation; aGVHD, acute graft-versus-host disease;

### The revised RMSD as a reliable algorithm for prospective prediction of aGVHD severity before allo-HSCT

Of the 20 evaluable recipient-donor pairs in the prospective study, there were 6 recipient-donor pairs who had 1 allele mismatching at either HLA-A or -B loci, 10 recipient-donor pairs who had 2 alleles mismatching at either HLA-A+B, -B+DRB1 or -DRB1+DQB1 loci, 2 recipient-donor pairs who had 3 alleles mismatching at HLA-A+B+DRB1 loci, and 2 recipient-donor pairs who had an HLA-haploidentical mismatching (Tables 2 and 3).

All of 20 recipients who entered this study underwent either related allo-HSCT or unrelated CB transplantation. As shown in Table 3, there was a strong correlation between single or total revised RMSD and the occurrence of severe aGVHD. The 6 recipient-donor pairs with 1 allele mismatching at either HLA-A or -B locus had a lower single revised RMSD (less than 0.50Å before allo-HSCT) and only I − II^0^ aGVHD occurred in these recipients after transplantation. All of recipient-donor pairs who developed severe aGVHD had a higher single or total revised RMSD (>0.50Å) before allo-HSCT, but not relevant to individual HLA-allele, allele group, loci, especially fixed combinations of different allele/loci. In addition, 2 recipient-donor pairs, P-UNP-01 and P-UNP-3, with an HLA-haploidentical mismatching had the highest total revised RMSD (1.4094Å and 1.2944Å) before allo-HSCT, and developed severe IV^0^ aGVHD after transplantation (Tables 2 and 3).

### The revised RMSD as a reliable algorithm for optimal donor selection

We further studied how to select optimal donor by HLA-TDSM system for 5 patients who had 2 − 6 alternative potential donors (Table 2 and 4). As shown in Table 4, P-UPN03 had 6 alternative potential cord blood donors with different 2 or 3 alleles mismatching at either HLA-A+DRB1 or -B+B or -A+B+DRB1 or -B+B+DRB1 loci, respectively. According to the guidelines for the minimum total revised RMSD < 0.50Å, CBU01 with 3 alleles mismatching at HLA-A+B+DRB1 loci was considered as the optimal donor owing to the minimum total revised RMSD (0.2580 Å) compared with those of CBU02 − 06, which had a revised RMSD of CBU02 − 06 0.4146Å, 0.5481Å, 0.6577Å, 0.5505Å, and 0.5047Å, respectively. CBU02 could be evaluated as the second choice donor due to total revised RMSD < 0.50Å. After transplantation with CBU01, only grade I aGVHD occurred in P-UPN03.

**Table 4:** The optimal donor-recipient selection based on HLA-TDSM system for recipients who had several alternative potential donors with comparably HLA allele-mismatched in 5 patient-donor pairs

UPN+	HLA alleles		Mismatches (MM)		Clinical status		RMSD (Å)	Revised RMSD (Å)	Total revised RMSD (Å)	aGVHD
	Patients	Donors	MM loci	No. of MM amino acids	Disease status	Transplant status				
**P-UPN08** 52/female	A*11: 01/30:01				AML/CR2	no				
	B*13: 01/38:01									
	DRB1* 07:01/12:02									
	DQB1* 02:01/03:01									
**Donor01** 56/brother		A*11: 01/30:01							1.3467	
		B*13: 01/27:04	B*38: 01/27:04	18			1.3510	1.3467		
		DRB1* 07:01/12:02								
		DQB1* 02:01/03:01								
**Donor02** 50/brother		A*11: 01/30:01							1.3467	
		B*13: 01/27:04	B*38: 01/27:04	18			1.3510	1.3467		
		DRB1* 07:01/12:02								
		DQB1* 02:01/03:01								
**Donor03** 47/brother		A*11: 01/30:01							1.3467	
		B*13: 01/27:04	B*38: 01/27:04	18			1.3510	1.3467		
		DRB1* 07:01/12:02								
		DQB1* 02:01/03:01								
**Donor04** 46/brother		A*24: 02/11:01	A*30: 01/24:02	25			0.4335	0.3847	3.4869	
		B*27: 04/27:04	B*13: 01/27:04 & 38: 01/27:04	23 & 18			1.3272 & 1.3510	1.3173 & 1.3467		
		DRB1* 12:02/12:02	DRB1* 07:01/12:02	19			0.3394	0.3023		
		DQB1* 03:01/03:01	DQB1* 02:01/03:01	18			0.1445	0.1359		
**P-UPN06** 39/female	A*11: 01/11:01				AML/CR1	yes				I^ö^
	B*18: 01/40:01									
	DRB1* 09:01/14:01									
	DQB1* 03:02/04:01									
Donor01 (*) 37/sister		A*11: 01/24:02	no MM in GVHD direction						0.1832	
		B*35: 01/40:01	B*18: 01/35:01	11			0.0864	0.0684		
		DRB1* 04:01/09:01	DRB1* 14:01/04:01	13			0.1186	0.1148		
		DQB1* 03:02/04:01								
**Donor02** 42/sister		A*11: 01/24:02	no MM in GVHD direction						1.0358	
		B*35: 01/15:02	B*18: 01/35:01 & 40: 01/15:02	11&19			0.0864& 0.5714,	0.0684& 0.4003		
		DRB1* 04:01/16:01	DRB1*09: 01/04:01&14: 01/16:01	16&13			0.3754& 0.2070	0.3663& 0.2008		
		DQB1* 03:02/04:01								
**Donor03** 45/sister		A*11: 01/11:01							0.7084	
		B*18: 01/15:02	B*40: 01/15:02	19			0.5714	0.4003		
		DRB1* 14:01/16:01	DRB1* 09:01/16:01	13			0.3933	0.3081		
		DQB1* 03:02/04:01								
**Donor04** 47/sisiter		A*11: 01/24:02	no MM in GVHD direction						0.1832	
		B*35: 01/40:01	B*18: 01/35:01	11			0.0864	0.0684		
		DRB1* 04:01/09:01	DRB1* 14:01/04:01	13			0.1186	0.1148		
		DQB1* 03:02/04:01								
**P-UPN03** 9/male	A*24: 02/33:01				ALL/CR1	yes				I^ö^
	B*15: 01/44:03									
	DRB1* 11:01/14:01									
	DQB1* 03:01/06:01									
Donor01 (*) Unrelated CB		A*11: 01/24:02	A*33: 01/11:01	15			0.3615	0.0930	0.2580	
		B*15: 01/44:02	B*44: 03/44:02	1			0.0730	0.0502		
		DRB1* 04:01/11:01	DRB1* 14:01/04:01	13			0.1186	0.1148		
		DQB1* 03:01/06:01								
**Donor02** Unrelated CB		A*24: 02/33:01							0.4146	
		B*44: 02/54:01	B*44: 03/44:02 & 15:01/54:01	1 &17			0.0730 & 0.6973	0.0502 & 0.2496		
		DRB1* 04:01/11:01	DRB1* 14:01/04:01	13			0.1186	0.1148		
		DQB1* 03:01/06:01								
**Donor03** Unrelated CB		A*24: 02/33:01							0.5481	
		B*44: 02/54:01	B*44: 03/44:02 & 15: 01/54:01	1 & 17			0.0730 & 0.6973	0.0502 & 0.2496		
		DRB1* 13:01/14:01	DRB1* 11:01/13:01	6			0.2698	0.2483		
		DQB1* 03:01/06:01								
**Donor04** Unrelated CB		A*11: 01/33:01	A*24: 02/11:01	24			0.4273	0.3774	0.6577	
		B*15: 01/44:02	B*44: 03/44:02	1			0.0730	0.0502		
		DRB1* 14:01/15:01	DRB1* 11:01/15:01	11			0.2474	0.2301		
		DQB1* 03:01/06:01								
**Donor05** Unrelated CB		A*24: 02/33:01							0.5505	
		B*18: 01/40:01	B*15: 01/18:01 & 44: 03/40:01	12 & 18			0.6855 & 0.5557	0.1941 & 0.3564		
		DRB1* 11:01/14:01								
		DQB1* 03:01/06:01								
**Donor06** Unrelated CB		A*33: 01/33:01	A*24: 02/33:01	24			0.5550	0.3899	0.5047	
		B*15: 01/44:03								
		DRB1* 04:01/14:01	DRB1* 14:01/04:01	13			0.1186	0.1148		
		DQB1* 03:01/06:01								
**P-UPN07** 18/male	A*02: 01/02:01				AML/CR1	yes				I^ö^
	B*13: 01/40:06									
	DRB1* 08:03/12:02									
	DQB1* 03:01/05:01									
Donor01 (*) Unrelated CB		A*02: 01/02:03	no MM in GVHD direction						0.1102	
		B*13: 01/40:01	B*40: 06/40:01	8			0.1325	0.1102		
		DRB1* 08:03/12:02								
		DQB1* 03:01/05:01								
**Donor02** Unrelated CB		A*02: 01/02:01							0.7125	
		B*13: 01/46:01	B*40: 06/46:01	23			0.5792	0.4151		
		DRB1* 08:03/12:02								
		DQB1* 03:01/06:01	DQB1* 05:01/06:01	15			0.3069	0.2974		
**P-UPN09** 20/female	A*02: 03/11:01				AML/CR1	no				
	B*38: 02/40:01									
	DRB1* 04:06/15:02									
	DQB1* 03:01/06:01									
Donor01 (*) Unrelated CB		A*11: 01/24:02	A*02: 03/24:02	22			0.1628	0.1532	0.1681	
		B*38: 02/40:01								
		DRB1* 04:05/15:02	DRB1* 04:06/04:05	4			0.0152	0.0149		
		DQB1* 03:01/06:01								
**Donor02** Unrelated CB		A*02: 06/11:01	A*02: 03/02:06	4			0.1188	0.1097	0.4725	
		B*40: 01/40:06	B*38: 02/40:06	18			0.5899	0.3556		
		DRB1* 04:06/15:01	DRB1* 15:02/15:01	1			0.0081	0.0072		
		DQB1* 03:01/06:01								
**Donor03** Unrelated CB		A*02: 06/11:01	A*02: 03/02:06	4			0.1188	0.1097	0.5101	
		B*15: 27/38:02	B*40: 01/15:27	17			0.5713	0.4004		
		DRB1* 04:06/15:02								
		DQB1* 03:01/06:01								

(*): optimal donor.

R-UPN, retrospective unique patient number; P-UPN, prospective unique patient number; MM, mismatches; RMSD, coordinate root-mean-square deviation; aGVHD, acute graft-versus-host disease; ALL, acute lymphoblastic leukemia; AML, acute myelocytic leukemia; CR, complete remission; CB, cord blood.

In contrast with P-UPN03, P-UPN06 had 4 alternative potential sibling donors (APSD) with either an HLA-B+DRB1 mismatching or an HLA-B+B+DRB1+DRB1 mismatching (Table 4). The total revised RMSD between P-UPN06 and each sibling APSD01 − 04 was 0.1832Å, 1.0358Å, 0.7084Å, and 0.1832Å, respectively. Thus, we preferred APSD01 with 2 alleles mismatching at HLA-B+DRB1 loci, the youngest sister of P-UPN06, as the optimal donor with the minimum total revised RMSD (0.1832Å) compared with other her sibling donors. After transplantation, only grade I aGVHD occurred in P-UPN06.

## DISCUSSION

Although the majority of recipients in need of allo-HSCT will lack fully HLA-matched related or unrelated donors, the vast majority of them will have available an HLA-mismatched related or unrelated donor [10, 12–20]. However, when faced with the majority of recipients having several alternative potential donors with 1 or more alleles mismatching at either single HLA-A/-B/-Cw/-DRB1/-DQB1 loci or different loci combinations, there is no clinical standard in place to determine which donor will produce the best transplantation.

This study is the first to show, to our knowledge, the successful development and application of a new selection/prediction system, HLA-TDSM, for optimal recipient-donor pair selection and accurate prediction of aGVHD severity in a prospective and retrospective randomized pilot clinical allo-HSCT study. HLA-TDSM selection/prediction system is based on the concept that any 3D structure changes at key sites (those influencing immunogenicity, peptide binding, and/or TCR interaction) of the mismatched HLA alleles may confer an increased risk of aGVHD after transplantation [29–31]. Our preliminary results clearly indicated that this system, which is based on single/total revised RMSD between recipient and donor, is a rapid, quantitative, and reliable algorithm by which transplantation physicians can quickly select the optimal donor-recipient pair from several alternative donors with 1 or more alleles mismatching at either single HLA-A/-B/-Cw/-DRB1/-DQB1 loci or different loci combinations, and even an HLA-haploidentical mismatching, prior to allo-HSCT.

Furthermore, based on the revised RMSD between any 2 alleles at each locus, we first theoretically defined all HLA acceptable versus unacceptable mismatched alleles and created a fast dictionary. Our preliminary results indicated that HLA acceptable versus unacceptable mismatched alleles at each locus are fixed and depend on the revised RMSD values between the 2 compared alleles at each locus. To our knowledge, this is the first clear-cut practical, qualitative, and quantitative dictionary involving HLA acceptable versus unacceptable mismatched alleles. The accurate prediction of aGVHD severity before allo-HSCT is critical for ensuring that recipients will undergo successful transplantation and be spared severe toxic effects of immunosuppressive therapy and treatment-related mortality. As yet, no validated biomarkers and/or exact prediction systems exist for the prediction and/or diagnosis of aGVHD severity before/after allo-HSCT, although several traditional prediction techniques, such as mixed lymphocyte culture (MLC), anti-recipient cytotoxic and helper T-lymphocyte precursor (CTLp/HTLp), panel-reactive T cells(PRT), and even multiple protein biomarkers and proteomic patterns, have been developed or described to date [31, 32]. These techniques have provided physicians with a few ability to predict the severity of aGVHD development prior to allo-HSCT.

Of crucial importance, HLA-TDSM system can also quickly and reliably predict aGVHD severity before allo-HSCT, which was confirmed by our prospective and retrospective randomized pilot clinical allo-HSCT study. Our preliminary results clearly demonstrated a strong correlation between single/total revised RMSD and aGVHD severity, except in R-UPN07, where 1 HLA-A/B loci recombination produced a novel haplotype. When single/total revised RMSD < 0.50Å, only grade I − II aGVHD will occur. In contrast, when single/total revised RMSD > 0.50Å, grade III − IV aGVHD will occur. All of these seem to be closely related to their 3D structure discrepancy of mismatched HLA alleles, but not the numbers and loci of mismatched HLA alleles. These results are not in agreement with the results of some recent studies, which demonstrated that aGVHD severity is likely related to the mismatched HLA loci and the total numbers of mismatched HLA alleles [12–18].

By comparison with past several computational matching algorithms such as cross-reactive groups (CREG), 3 residual matching (TSM), HLAMatchmaker, and HistoCheck, all of which were only limited in the theory but not practically used in clinical allo-HSCT owing to their rather contradictory results [33–36], HLA-TDSM system has mainly focused on several aspects of progression from protein 3D structures modeling, HLA matching/mismatching technique, computational biology, bioinformatics, transplantation immunology, and an extensive larger-scale cohort clinical allo-HSCT experience. Its theoretical and clinical implications are as follows: 1) Whatever alleles or proteins that are identical either in antigen-recognition site domain or in 3D structures have been shown to have the similar and/or even same immunologic or biological functions [29, 30]. 2) The polymorphism of AAR of both HLA class I and II molecules with some similar characteristics is mainly focused on the region of antigen-binding groove that is alpha helix and beta sheet. Types and frequency of AAR varied greatly on different positions of HLA polypeptide. On most of the positions, there are only 2 or 3 possible AAR types with 1or 2 types in dominant [29, 30]. 3) There exist seemingly some different degrees of acceptable/unacceptable HLA mismatched alleles/antigens at each locus [8–20, 27, 28], though there is currently no evidence to unequivocally demonstrate which acceptable/unacceptable HLA mismatched alleles/antigens should be preferred in any clinical circumstance.

In conclusion, we successfully developed and applied a new selection/prediction system based on HLA-TDSM discrepancy for prediction of aGVHD severity and selection of optimal donor-recipient pairs in a prospective and retrospective randomized pilot clinical allo-HSCT study. We believe that HLA-TDSM system is essential not only for optimal donor-recipient selection before allo-HSCT, but also for the refinement of pre-emptive therapy on aGVHD after allo-HSCT.

## MATERIALS AND METHODS

### Patient and donor characteristics

This study of prospective prediction and retrospective verification of aGVHD severity by HLA-TDSM system was based in the Beijing 307 Hospital and the protocol was approved by the Academy of Medical Sciences Review Board. Informed consent was obtained from all patients and donors or their guardians in accordance with the Declaration of Helsinki.

Thirty-seven patient-donor pairs from several treatment centers were evaluated from January 2002 to August 2010, comprising 33 sibling and 4 unrelated recipient-donor transplant pairs. Of these, 25 pairs were prospective (5 patients did not complete the transplant owing to either disease deterioration or economic constraints) and 12 pairs were retrospective. Of the 32 pairs who completed the transplant, 12 had 1 HLA-allele mismatch, 13 had 2 HLA-allele mismatches, 5 had 3 HLA-allele mismatches, and 2 were haploidentical at the HLA allele. Of the 32 transplant recipients, 5 had 2 − 6 alternative potential donors with comparable HLA-allele mismatch to be selected respectively. For 3 of these recipients, the alternates were unrelated donors from a cord blood donor bank, while for the other 2 recipients, the alternates were sibling donors. Both recipient and donor details are summarized in Table 2.

### HLA typing at low-resolution and sequence-based typing

The patients and donors were typed at low- and high-resolution levels by PCR-SSP and sequence-based typing methods, respectively, for HLA-A/-B/-DRB1/-DQB1 loci as previously described [31].

### GVHD prophylaxis and evaluation

Patients were prepared for transplantation with the use of standard myeloablative conditioning regimens and all patients received cyclosporine (CsA) +methotrexate (MTX) as aGVHD prophylaxis as previously described [37]. aGVHD was diagnosed and graded according to established clinical criteria [1, 10].

### HLA 3D structure modeling and database management system

The 3D structures of all alleles at HLA-A/-B/-Cw/-DRB1/-DQB1/-DPB1 loci were modeled individually (for details, see the Supplementary Appendix).

### Calculation of the coordinate RMSD and revised RMSD between HLA-alleles at all loci

The coordinate RMSD is frequently used to measure the differences between values predicted by a model or an estimator and the values actually observed [38, 39]. In our study, the revised RMSD, a corrected value of coordinate RMSD, was used to evaluate the structure discrepancy degree between any 2 HLA-alleles at each loci according to the structural features of HLA class I and II molecules.

Additional methods on HLA 3D structure modeling and data base management system; construction, transfection, expression, and characterization of pcDNA3.1/HLA-B eukaryotic expression vectors; measurement of serum anti-EBNA antibody and affinity analysis between EBNA3 nonapeptide and HLA molecules; induction of antigen-specific alloreactive CTLs; TCR Vβ gene scan; flow cytometric analysis; proliferation and cytotoxicity assays; statistical analysis; and associated references are provided in the Supplementary Appendix.

## SUPPLEMENTARY MATERIALS AND METHODS, FIGURES AND TABLES


