# Supplementary material for: Three-dimensional structure discrepancy between HLA alleles for effective prediction of aGVHD severity and optimal selection of recipient-donor pairs: a proof-of-concept study
Source: Oncotarget. 2015 Oct 16;6(37):40337–59. doi: 10.18632/oncotarget.5378 (PMC4741899; doi:10.18632/oncotarget.5378)
Supplement: Supplementary file 1 [file oncotarget-06-40337-s001.pdf]

SUPPLEMENTARY MATERIALS AND METHODS

HLA 3D structure modeling and database management system

The 3D structures of all alleles at HLA-A/-B/-Cw/-DRB1/-DQB1/-DPB1 loci were modeled one by one. The amino acid sequences of all HLA-alleles were obtained from IMGT/HLA database (http://www.ebi.ac.uk/imgt/hla/), which provided a centralized repository for the sequences of the alleles named by the WHO Nomenclature Committee for Factors of the HLA System [1]. The 3D structural models of HLA proteins were constructed with Swiss-PdbViewer software and the SWISS-MODEL automated homology modeling server [2–8].

Although α heavy chains at HLA-I class molecular have multiple structures, only α1 and α2 chains of extracellular polypeptide-binding domains containing 180 amino acids participate in antigen binding site. So we have modeled the molecular structures of these 180 amino acids. Due to only β chain at HLA-DR locus from HLA-II class molecular having polymorphism (α chain with fewer polymorphism), which is at extracellular polypeptide-binding domains containing 90 amino acids so that we have only modeled the molecular structures of these 90 amino acids. In contrast, both α and β chains at HLA-DQ/DP loci from HLA-II class molecular have polymorphism, thus, the molecular structures of both α and β chains of extracellular polypeptide-binding domains amino acids were modeled.

The results were managed with Microsoft Office Access software, and a database management tools-HLAStrucMark system was compiled with by VC, received the software copyright registration certificates in 2007(066945) [5]. With this system, detailed information about mismatched amino acid residues between any two HLA molecules can be shown such as the mismatched amino acid residues position, the function characters of the position, anchoring residues, contributing to peptides presenting, and TCR binding, et al.

Calculation of the coordinate RMSD and revised RMSD between HLA-alleles at all loci

RMSD is a frequently-used measure of the differences between values predicted by a model or an estimator and the values actually observed from the thing being modeled or estimated [9, 10]. These individual differences are also called residuals, and the RMSD serves to aggregate them into a single measure of predictive power. All of HLA alleles are similar within locus, having the same amino acid number, similar domains, similar super-secondary structure (motif) [1]. In our study, the coordinate RMSD was employed to evaluate the degree of structure differences of HLA alleles. Firstly, the centroid of two HLA proteins were superimposed, and then backbone atoms including alpha carbon atoms , carbonyl carbon atoms and nitrogen atoms were selected to calculate RMSD parameters [11–15].

The RMSD parameters were calculated by Visual Molecular Dynamics (VMD, http://www.ks.uiuc.edu/research/vmd), which was aided by National Institutes of Health(NIH), developed by Beckman institute of University of Illinois at Urbana-Champaign, and used for biophysics computing of protein, nucleic acid, lipid et. al. VMD software can be programmed, scripts were used to compute the RMSD of HLA-alleles high-throughputly and accurately. RasMol is developed by R. Sayle in the University of Edinburgh for the visualization of biomacromolecule, which can deal with the atomic coordinate data. In our study, RasMol was used to visualize the structure differences of HLA -alleles between patient-donor pairs in PC [3–7].

Due to the facts that amino acid residues (AAR) differences at key site between mismatched HLA-alleles at each HLA locus can changed the polarity and hydrogen bonding of the site, thus, some important 3D structures made up of either the altered amino acid itself or the nearby residues, for peptide binding and/or TCR interaction, were significantly changed through alterations to van der Waals forces, short-range interaction and/or long-range interaction [16]. AAR diversity at HLA class I and II molecules are mainly focused on the region of antigens binding groove which are consisted of 2 alpha helix and 8 beta sheet, especially in the regions of the first alpha helix and beta sheet which are extremely important for TCR binding and antigen peptide presenting (Tables 1–4 and Figure 1A–1B in the Supplementary Appendix). As we all know that not every AAR participate in antigen peptide presenting or TCR binding especially AAR in region of random coil, but all these AAR were still involved in the coordinate RMSD calculation. For example,when calculating the coordinate RMSD between HLA-B*07:13 and HLA-B*08:09 allleles, AAR in random coil region such as residues 39–44 (Figure 2 in the Supplementary Appendix) will be greatly affectting the last calculated results. Thus, The parameters of RMSD calculation were revised according to the structure features of HLA alleles, and the amino acids residues located in α1/α2 domain at HLA I or α1 domain at HLA II interacting with neither peptide nor TCR were excluded in the calculation of the revised RMSD [17–20].

Construction, transfection, expression and characterization of pcDNA3.1/HLA-B eukaryotic expression vector

The full-length cDNA coding for HLA-B alpha chain was synthesized from the donor's blood cells (RNA LA PCR kit, TaKaRa Bio, Otsu, Shiga, Japan), ligated with PGEM-T-easy vector and then cloned

into the pcDNA3.1/Zeo(+) expression vector (In Vitrogen, Leek, The Netherlands), Restriction enzyme sites (*HindIII* and *EcoRI*) were designed into primers (shown in bold type): sense:5′-GGGCAAGCTTGGACT CAGAATCTCCCCAGACGCC GAG-3′, antisense: 5′-GCCGGAATTCCCCTCACAAGACAGCTGTCTCAG GC-3′. The resulting recombinant plasmid, pcDNA3.1/HLA-B was confirmed by sequencing, produced in E. coli and purified with a PureYield plasmid midiprep system (Promega, Madison, WI, USA), and used as the eukaryotic expression vector in this study (Figures 4a–4e in the Supplementary Appendix).

pcDNA3.1/HLA-B was transfected into Hmy2.CIR cell line using Amaxa Nucleofector System, that is both HLA-A and HLA-B protein defective immortalized B cell line (Cell Bank of Chinese Academy of Sciences, Beijing, China), and selected by Zeocin. Enhanced Green Fluorescent Protein (EGFP) was used as positive control. Total RNA was extracted from stable transfected Hmy2.CIR–HLA-B cells (TRIzol reagent; Invitrogen, USA), and HLA-B cDNA was amplified (RNA LA PCR kit; TaKaRa Bio, Otsu, Shiga, Japan) a 1086-bp fragment. Human beta-actin cDNA (650 bp) was amplified as the control. The expressed HLA-B protein in transfected Hmy2.CIR cells was identified by flow cytometric analysis, immunohistochemical examination and Western-blot respectively as previously described (Figures 5a–5g in the Supplementary Appendix) [21, 22].

Measurement of serum Anti-EBNA antibody and affinity analysis between EBNA3 nonapeptide with HLA molecules

The levels of serum antibodies to Epstein-Barr virus nuclear antigen (EBNA3) from healthy volunteer blood were measured by ELISA (EUROIMMUN, AG, GEM) according to the manufacture's instruction. There are lots of methods for calculation of the affinity between molecules, and the prediction of the Interaction of peptide-MHC based on molecule docking algorithm to get the conformation in the lowest free energy state was popular. To avoid the system deviation, three different algorithm: NetMHC Server, MMBPred, and SYFPEITHI were employed to evaluate the affinity between EBNA3 nonapeptide with HLA molecules as previously described. The three methods were all based on net server and convenient to use peptides and HLA allele were submitted properly, and the feedbacks would be given with internet.

Induction of antigen-specific alloreactive CTLs *in vitro*

The synthesized EBNA3 nonapeptide (FLRGRAYGL) was used as stimulating antigens and its purity was greater than 95% determined by mass spectrometry and high-performance liquid chromatography. Stock solutions of EBNA3 nonapeptide solubilized in dimethyl sulfoxide (DMSO) were kept at −70°C. The transfected Hmy2.CIR cells with either pcDNA3.1 HLA-B*1502 or -B*1518 or -B*3503 or -B*4403 allele pulsed with EBNA3 nonapeptide, were used as APC cells to induce antigen-specific alloreactive CTLs *in vitro* respectively. Peptide pulsing was performed for 1 hour at room temperature at a final concentration of 10 μmol/L and washed twice. Mitomycin C (Kyowa Hakko Kogyo Inc., Japan) treating was performed for 30 min at room temperature at a final concentration of 30 μg/ml and washed twice. The antigen-specific alloreactive CTLs was induced *in vitro* as previously described. Briefly, human purified T cells were isolated from heparinized peripheral blood of healthy EBV-seropositive individuals as previously described. For the generation of EBV-specific CTLs, the following stimulation procedures were used. Firstly, purified T cells in RPMI 1640 supplemented with 10% heat-inactivated human AB serum were incubated in 12-well plates (Costar) at a final concentration of 2 × 106 cells/well with 2 × 104 stimulator cells prepared already. And then, hrIL-2 (50U/ml, Quangang Pharmaceutical Co., Ltd, Shandong,China), mouse anti-human CD3 mAb (1 μg/mL, Abcam Inc., UK), hrIL-4 (30 ng/ml, Sigma Inc., USA) and PHA-P (5 μg/mL, Sigma Inc., USA) were supplemented after 24 hrs. On days 3 and 7 of cultures, each well received 1 × 105 stimulator cells, and cultures were fed every 3–4 d with RPMI 1640 completed medium containing hrIL-2, anti-CD3 mAb ,rhIL-4 and PHA-P. So like this cultured T cells were harvested after 14 days and used for EBV-specific CTLs *in vitro* experiments.

TCR Vβ gene scan analysis

Total RNA was prepared from the transfected Hmy2.CIR cells and cDNA was synthesized by reverse transcriptase PCR (RT-PCR) with an oligo dT primer (RNA LA PCR kit, TaKaRa Bio, Otsu, Shiga, Japan). Human Glyceraldehyde 3-phosphate dehydrogenase (GAPDH) cDNA (350 bp) was amplified as the control. For TCR Vβ family-specific amplification, PCR was performed using 24 Vβ family-specific primers and two anti-sense primers binding in the TCR Vβ C region primer (5′ fluorescently labeled with Fam and Hex respectively). PCR products of about a 255 bp fragment (range 240–290 bp) were generated on a 2.5% agarose gel. For TCR Vβ gene sequence analysis, 1 μl of PCR product was mixed with 25 μl formamide and 0.3 μl of the internal size standard Genescan-2500 Rox (Applied Biosystems, Warrington, UK). PCR products were denatured for 5 min at 95°C and size separated on a high resolution polyacrylamide gel on the ABI automated gene sequencer (ABIPRISM 310 Genetic Analyzer) and analyzed using the GENESCAN software.

Fluorescent PCR products are represented as electrophoregrams where relative fluorescence intensities are plotted as a function of PCR fragment size. Products from clonal cell populations were visualized as one or two sharp peaks of fluorescence corresponding to the PCR amplified clonal rearranged alleles, while cDNA extracted from normal polyclonal peripheral blood cells was visualized as a fluorescence spectrum composed of polyclonal PCR fragments of different sizes in a normal Gaussian distribution with peaks spaced by 3 bp corresponding to selected in-frame rearrangements. Oligo-clonal profiles were visualized as a modification of the Gaussian distribution where three or more distinct peaks appeared above the normal polyclonal background.

Flow cytometric analysis of $CD4^+$and $CD8^+$ T cells

The induced CTLs were harvested and different subsets were determined by flow cytometric assay after induced 14 days by the transfected Hmy2.CIR cells as previously described [21, 22]. Briefly, the induced CTL and uninduced cells were incubated with various monoclonal antibodies including FITC-anti-human CD3, PerCP-anti-human CD8 and PE-anti-human CD4 (Becton Dickinson, USA) for 1 h at RT in the dark. After three washes with PBS, the cells was resuspended in 500 μl PBS/2% paraformaldehyde solution and analyzed by FACSCalibur instrument and CellQuest software.

Proliferation assays of the induced CTLs

To investigate the proliferation inhibitory activity of the induced CTLs, the transfected Hmy2.CIR cells pulsed with EBNA3 nonapeptide on 96-well plates (2×10^4/ml) were co-cultured with the induced CTLs cells (1×10^5/ml) for 3 hrs at 37°C in 5% CO2 air as previously described [21, 22]. CellTiter 96® Aqueous One Solution Cell Proliferation Assay kit (Promega, Madison, WI, USA) was used to detect the viable cells in the co-culture. After 3 hrs incubation, the O.D. at 570 nm was measured with an ELISA plate reader. The proliferation was determined as follows: (viable cells in the killer and target cell co-culture/numbers of viable cells in the control and target cell co-culture) × 100%.

Cytotoxicity of the induced CTLs

To investigate cytotoxicity of the induced CTLs, the transfected Hmy2.CIR cells with pulsed with EBNA3 nonapeptide on 96-well plates (2×10^4/ml) were co-cultured with the induced CTLs cells (1×10^5/ml) for 3 hrs at 37°C in 5% CO_2 air as previously described [21, 22]. The transfected Hmy2.CIR cells, the induced CTLs, the transfected Hmy2.CIR cells treated with Lysis Solution, 1640 complete medium, and 1640 complete medium treated with Lysis Solution were used as target cell spontaneous LDH release control, effector cell spontaneous LDH release control, target cell maximum LDH release control, culture medium background control, and volume correction control respectively. CytoTox 96® Non-Radioactive Cytotoxicity Assay kit (Promega, Madison, WI, USA) was used to detect Lactate Dehydrogenase (LDH) quantitatively that released upon cell lysis. After 3 hrs incubation, 50 μl culture supernatants were collected and added with 50 μl Cytotoxicity Assay Buffer in enzymatic assay plate for 30 mins at RT in the dark. The O.D. at 570 nm was measured with an ELISA plate reader. The proliferation was determined as follows: Cytotoxicity(%) = (Experimental-Effect Spontaneous-Target Spontaneous)/(Target Maximum-Target Spontaneous) × 100%

Study patient-donor pairs

We studied 37 patient-donor pairs from several treatment centers were evaluated, comprising 33 sibling and 4 unrelated recipient-donor transplant pairs. Of these, 25 pairs were prospective (5 patients did not complete the transplant owing to either disease deterioration or economic constraints) and 12 pairs were retrospective. Of the 32 pairs who completed the transplant, 12 had 1 HLA-allele mismatch, 13 had 2 HLA-allele mismatches, 5 had 3 HLA-allele mismatches, and 2 were haploidentical at the HLA allele. Of the 32 transplant recipients, 5 had 2-6 alternative potential donors with comparable HLA-allele mismatch to be selected respectively. For 3 of these recipients, the alternates were unrelated donors from a cord blood donor bank, while for the other 2 recipients, the alternates were sibling donors. Both recipient and donor details are summarized in Table 2.

This study of prospective prediction and retrospective verification of aGVHD severity by HLA-TDSM system was based in the Beijing 307 Hospital and the protocol was approved by the Academy of Medical Sciences Review Board. Informed consent was obtained from all patients and donors or their guardians in accordance with the Declaration of Helsinki.

HLA typing at low-resolution and sequence-based typing

The patients and donors were typed at low- and high-resolution levels by PCR-SSP and sequence-based typing methods, respectively, for HLA-A/-B/-DRB1/-DQB1 loci as previously described.

GVHD prophylaxis and evaluation

Patients were prepared for transplantation with the use of standard myeloablative conditioning regimens and all patients received cyclosporine(CsA)+methotrexate

(MTX) as aGVHD prophylaxis as previously described [23]. aGVHD was diagnosed and graded according to established clinical criteria [24, 25].

Statistical analysis

Data were analyzed using the SPSS11.5 software program (SPSS, USA). Group comparisons were performed using the chi-square test or the Mann-Whitney test for independent samples (non-parametric data). Probability (*P*) values < 0.05 were considered significant.

REFERENCES

1. IMGT/HLA . . http://www.ebi.ac.uk/imgt/hla/align.huml.
2. Ren EC, Kangueane P, Kolatkar P, Lin MT, Tseng LH, Hansen JA. Molecular modeling of the minor histocompatibility antigen HA-1 peptides binding to HLA-A alleles. Tissue Antigens. 2000; 55:24–30.
3. Peitsch MC. ProMod and Swiss-Model: Internet-based tools for automated comparative protein modelling. Biochem Soc Trans. 1996; 24:274–279.
4. Peitsch MC, Jongeneel V. A 3-dimensional model for the CD40 ligand predicts that it is a compact trimer similar to the tumor necrosis factors. Int Immunol. 1993; 5:233–238.
5. Peitsch MC. ProMod: automated knowledge-based protein modelling tool. PDB Quarterly Newsletter. 1995; 72:4–12.
6. Peitsch MC. Protein modelling by E-Mail. Biotechnology. 1995; 13:658–667.
7. Peitsch MC, Wilkins MR, Tonella L, Sanchez JC, Appel RD, Hochstrasser DF. Large scale protein modelling and integration with the SWISS-PROT and SWISS-2DPAGE databases: the example of Escherichia coli. Electrophoresis. 1997; 18:498–501.
8. Xi YZ, Sun YY, Yuan F, Luo WD. The scoring system for 3-dimensional structure matching of human leukocyte antigens—-HLAStrucmark. Computer software copyright registration certificate authorized by national copyright administration of P.R.China, Registration No.2007SR00950.
9. Jewett AI, Huang CC, Ferrin TE. MINRMS: an efficient algorithm for determining protein structure similarity using root-mean-squared-distance. Bioinformatics. 2003; 19:625–634.
10. Maiorov VN, Crippen GM. Significance of root-mean-square deviation in comparing three-dimensional structures of globular proteins. J Mol Biol. 1994; 235:625–634.
11. Bates PA, Sternberg MJ. Model buiding by comparison at CASP3: using expert knowledge and computer automation. Proteins. 1999; :47–54.
12. Burke D, Deane CM, Nagarajaram HA, Campillo N, Martin-Martinnez M, Mendes J, Molina F, Perry J, Reddy BV, Soares CM, Steward RE, Williams M, Carrondo MA, et al. An iterative structure assisted approach to sequence alignment and comparative modeling. Proteins. 1999; :55–60.
13. Peitsch MC. Protein modeling by E-mail. From amino acid sequence to protein structure: a free one-hour service. Biotechnology. 1995; 13:658–664.
14. Moult J, Hubbard T, Fidelis K, Pedersen JT. Critical assessment of methods of protein structure prediction (CASP): round III. Proteins. 1999; 3:2–6.
15. Hubbard TJP. RMS/coverage graphs: a qualitative method for comparing three dimensional protein structure predictions. Proteins. 1999; 3:15–21.
16. Sun YY, Kong F, Ren S, Yuan F, Liang F, Liu N Jin L, Xi YZ. Severe acute graft-vs-host disease in a patient with acute monocytic leukemia having a recombination event between HLA-A/B loci from a multiple recombinant family. Tissue Antigen. 2007; 70:499–505.
17. Meng WS, Grafenstein H, Haworth IS. A model of water structure inside the HLA-A2 peptide binding groove. Int Immunol. 1997; 9:1339–1346.
18. Garboczi DN, Ghosh P, Utz U. Structure of the complex between human T-cell receptor, viral peptide and HLA-A2. Nature. 1996; 384:134–141.
19. Smith KJ, Pyrdol J, Gauthier L, Wiley DC, Wucherpfennig KW. Crystal structure of HLA-DR2 (DRA*0101, DRB1*1501) complexed with a peptide from human myelin basic protein. J Exp Med. 1998; 188:1511–1520.
20. Dessen A, Lawrence CM, Cupo S, Zaller DM, Wiley D. X-ray crystal structure of HLA-DR4 (DRA*0101, DRB1*0401) complexed with a peptide from human collagen II. Immunity. 1997; 7:473–481.
21. Song X, Liang F, Liu N, Luo Y, Xue H, Yuan F, Tan L, Sun Y, Xi C, Xi Y. Construction and characterization of a novel DNA vaccine that is potent antigen-specific tolerizing therapy for experimental arthritis by increasing CD4+CD25+Treg cells and inducing Th1 to Th2 shift in both cells and cytokines. Vaccine. 2009; 27:690–700.
22. Xue H, Liang F, Liu N, Song X, Yuan F, Luo Y, Zhao X, Long J, Sun Y, Xi Y. Potent Antirheumatic Activity of a New DNA Vaccine Targeted to B7–2/CD28 Costimulatory Signaling Path-way in Autoimmune Arthritis. Human Gene Therapy. 2011; 22:65–76.
23. Kanda Y, Izutsu K, Hirai H, Sakamaki H, Iseki T, Kodera Y, Okamoto S, Mitsui H, Iwato K, Hirabayashi N, Furukawa T, Maruta A, Kasai M, et al. Effect of graft-versus-host disease on the outcome of bone marrow transplantation from an HLA-identical sibling donor using GVHD prophylaxis with cyclosporin A and methotrexate. Leukemia. 2004; 18:1013–1019.
24. Gooley TA, Chien JW, Pergam SA, Hingorani S, Sorror ML, Boeckh M, Martin PJ, Sandmaier BM, Marr KA, Appelbaum FR, Storb R, McDonald GB. Reduced mortality

after allogeneic hematopoietic-cell transplantation. N Engl J Med. 2010; 363:2091–2101.

25. Shaw PJ, Kan F, Woo Ahn K, Spellman SR, Aljurf M, Ayas M, Burke M, Cairo MS, Chen AR, Davies SM, Frangoul H, Gajewski J, Gale RP, et al. Outcomes of pediatric bone marrow transplantation for leukemia and myelodysplasia using matched sibling, mismatched related, or matched unrelated donors. Blood. 2010; 116:4007–4015.

SUPPLEMENTARY FIGURES AND TABLES

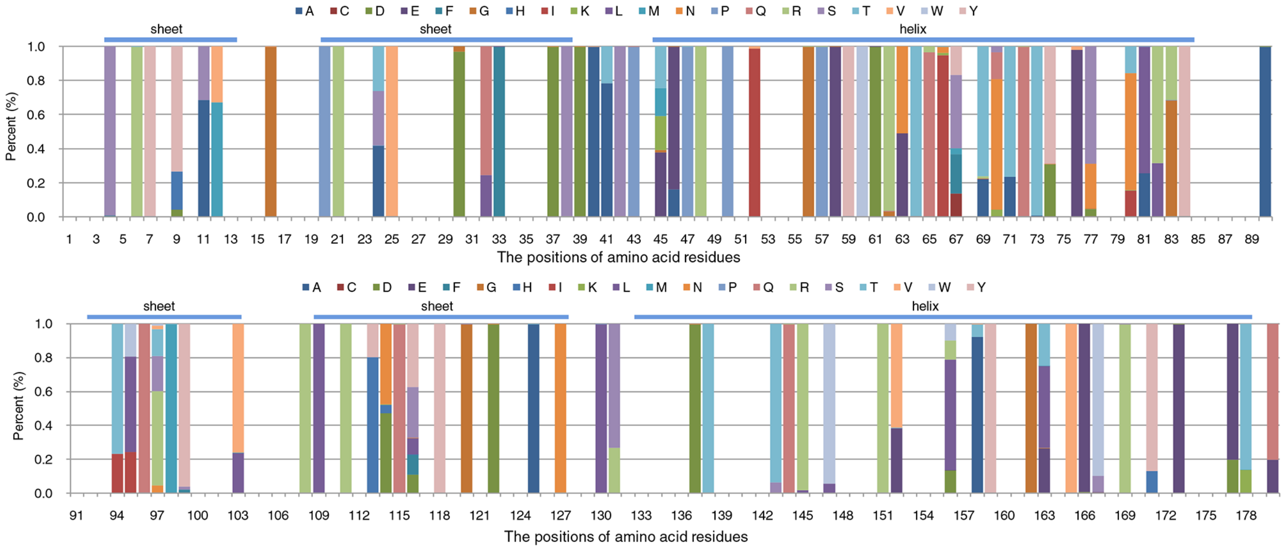

Supplementary Figure S1a: Amino acid residues polymorphism of HLA-B molecules.

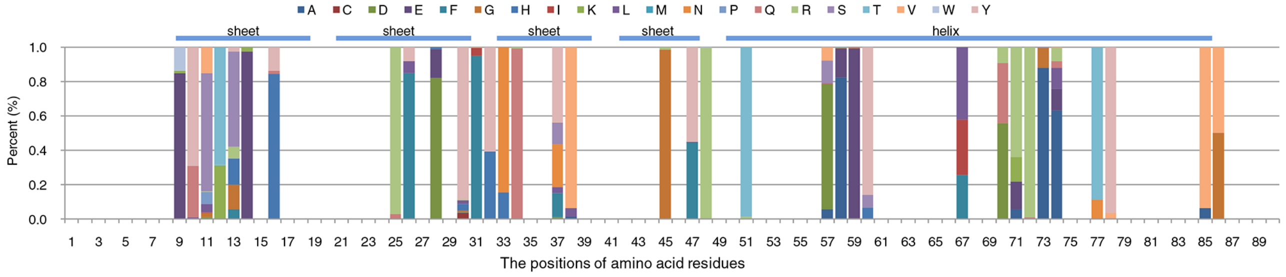

Supplementary Figure S1b: Amino acid residues polymorphism of HLA-DRB1 molecules. HLA-B and HLA-DRB1 loci were selected as representative of HLA-I/II class moleculars. The polymorphic distribution of amino acid residues 1 □ 180 from HLA-B and amino acid residues 1 □ 90 from HLA-DRB1(1b) were showed in figures 1a and 1b respectively. The position of amino acid residues was showed on horizontal abscissa and the different amino acid residues and their contents were presented with different colors and lengths. English capital letters display different amino acids respectively.

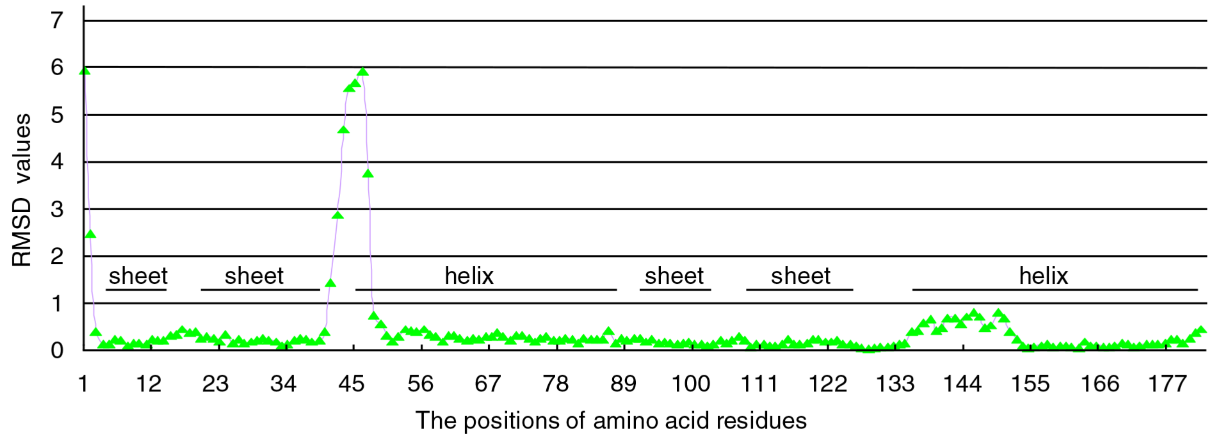

Supplementary Figure S2: The effect of amino acid residues in un-function region to RMSD calculation. The comparison between HLA-B*07:13 and HLA-B*08:09 alleles. The position of amino acid residues were showed on horizontal abscissa and the function region were presented with black bar.

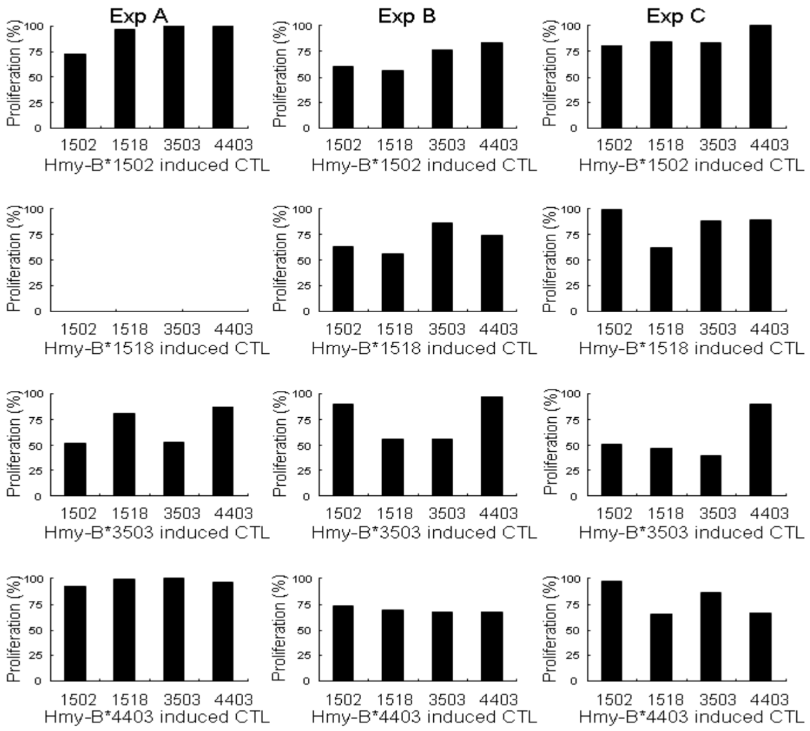

Supplementary Figure S3a: proliferation assays of CTLs.

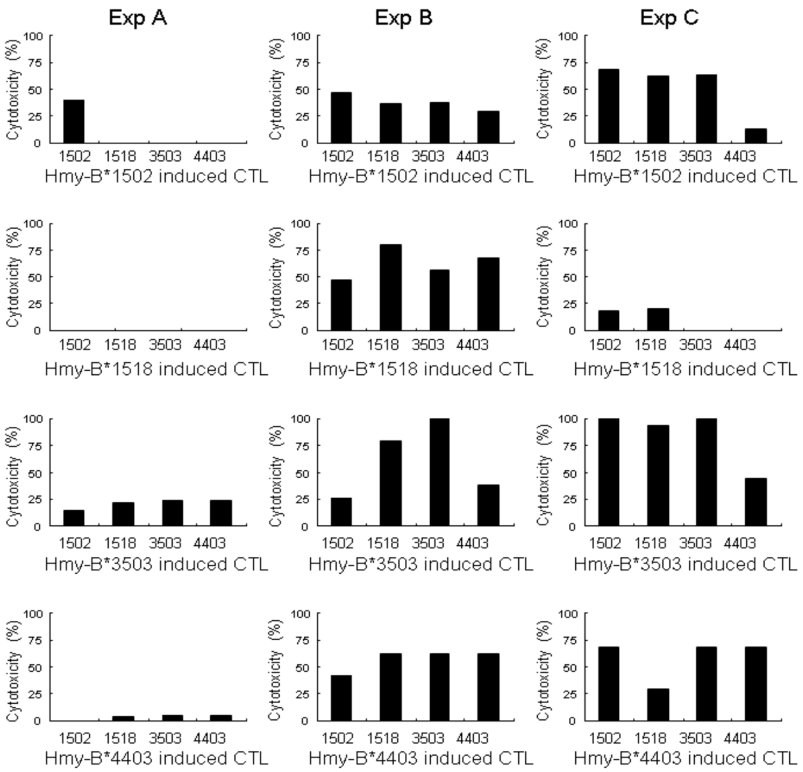

Supplementary Figure S3b: cytotoxicity assays of CTLs. The results showed that CTLs induced by non-natural low affinity binding MHC-Pep complex had lower ability on inhibiting proliferation. The results showed that the CTLs induced by non-natural MHC-Pep with low affinity had weak ability of inhibit proliferation. As shown in figyre 7a, induced CTLs origin from volunteer Exp B, which should be HLA-B*51:01 or HLA-B*67:02 restricted initially, exhibited weak inhibit proliferation ability to all the target Hmy2.CIR expressing either HLA-B*15:02, HLA-B*15:18, HLA-B*35:03 or HLA-B*44:03. HLA-B*35:03-EBNA3 induced CTLs origin from volunteer Exp C exhibited efficient inhibition of proliferation (up to 39%), while reacted with target Hmy2.CIR expressing HLA-B*35:03. We observed obvious cross-reaction on the inhibiting effect of proliferation for CTLs induced by non-self MHC-Pep: HLA-B*44:03-EBNA3 induced CTLs origin from volunteer Exp B had a general inhibiting effect on target Hmy2.CIR expressing HLA-B*15:18,HLA-B*35:03 and HLA-B*44:03 by the ratio of 69.6%, 67.3% and 67.1% separately. The cytotoxicity of CTLs for allogenetic HLA was also analyzed as show in Figure 7b, and the results showed more susceptibility than the proliferation inhibition analysis. The induced CTLs showed much different killing ability. For example, HLA-B*35:03-EBNA3 induced CTLs showed 100% cytotoxicity to target Hmy2.CIR expressing the same HLA molecule, while HLA-B*15:18-EBNA3 induced CTLs origin from volunteer Exp B showed only 20% killing rate. The cross cytotoxicity was observed on the reaction with allogenetic HLA-B*44:03-EBNA3 induced CTLs origin from volunteer Exp B had cytotoxicity on target Hmy2.CIR cells expressed different HLA, which acted similar with the results of proliferation assay. While HLA-B*35:03-EBNA3 induced CTLs origin from volunteer Exp B only reacted with target Hmy2.CIR cells expressed HLA-B*35:03 and HLA-B*15:08, but not HLA-B*44:03. HLA-B*35:03-EBNA3 induced CTLs origin from volunteer Exp C exhibited the similar characters with the CTLs origin from volunteer Exp B.

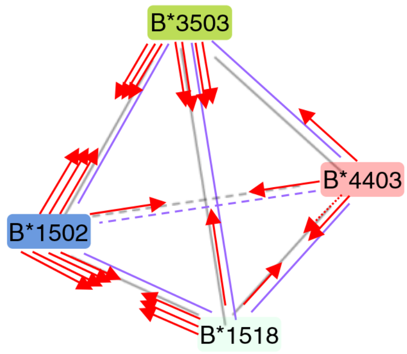

	Rmsd	Rmsd revised	SSM
B*1502-B*1518:	0.70	0.20	5.38
B*1502-B*3503:	0.70	0.20	6.25
B*1502-B*4403:	0.36	0.34	12.76
B*1518-B*3503:	0.10	0.07	6.94
B*1518-B*4403:	0.80	0.40	15.17
B*3503-B*4403:	0.80	0.40	13.77

Supplementary Figure S5a: The correlation between allogeneic cross-reaction patterns by the induced antigen-specific alloreactive CTLs *in vitro* and HLA3D structure discrepancy. Proliferation inhibition or cytotoxicity of the induced CTLs origin from Exp A were low in general, while HLA-B*15:02 induced CTLs identified specifically and HLA-B*35:03 induced CTLs exhibited cross-reaction obviously. Cross-reaction were existed commonly of the induced CTLs origin from Exp B, reflected as HLA-B*15:02 induced CTLs cross-reaction to HLA-B*35:05, HLA-B*35:05 induced CTLs cross-reaction to HLA-B*15:02 and HLA-B*15:18. As to the induced CTLs origin from Exp C, cross-reaction were displayed as HLA-B*35:05 induced CTLs cross-reaction to HLA-B*15:02 and HLA-B*15:18. In our study, transfected Hmy2.CIR cells pulsed with a nonameric consensus peptide (EBNA3) were used as target cells and inducing cells in the vitro experiments, and HLA antigens were the only variable factors in TCR-MHC-Pep. And then the cross-reaction pattern of induced CTLs could reflect the function differences of HLA antigens indirectly.

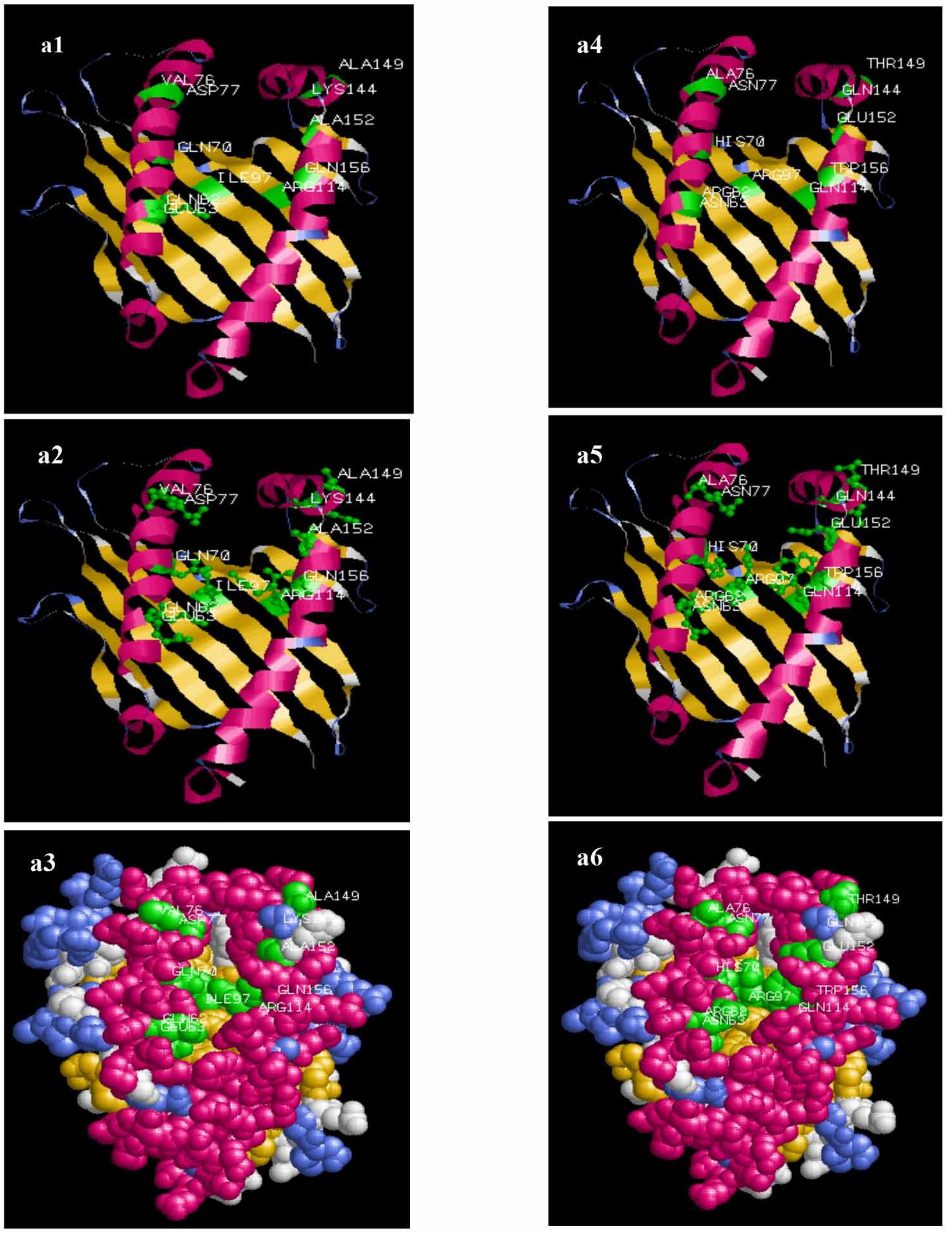

Supplementary Figure S5a: Eight representative of three-dimensional structure modeling analysis for each mismatched HLA-A/-B/-DRB1/-DQB1 alleles on allo-HSCT recipient-donor pairs from either retrospective or prospective studies. Using RASMOL software version 2.7.5., the structure of the a1 and a2 domains is colored using RASMOL 'structure' color scheme that colors the molecule by protein secondary structure. Alpha helices are colored magenta, beta sheets are colored yellow, turns are colored pale blue and all other residues are colored white. Mismatched amino acids in each structure are colored green and labeled white. **In figure 5a.** a1-a3 and a4-a6 represent three-dimensional structure modeling analysis of the a1 and a2 domains for HLA-A*11:01/A*26:01 of P-UPN14 donor-recipient pair, respectively. The position and structure of 10 mismatched amino acids are displayed in a1/a2 and a4/a5 for HLA-A*11:01 and A*26:01, respectively. The a3 and a6, the representation all the atoms displayed as solid spheres, showing the union-of-spheres models for HLA-A*11:01 and A*26:01 are displayed in a3 and a6, respectively. The amino acids 62, 63,70,76,77,97, 114,144,149,152 and 156 are the different AARs between HLA-A*11:01/A*26:01. From figure a1 to a6, it can be clearly observed that the structural differences between the mismatched AARs in peptide-binding sites or TCR interaction positions were far smaller with only 0.0403Å of total revised RMSD value, therefore only grade I aGVHD occurred in P-UPN14 after transplantation. (*Continued*)

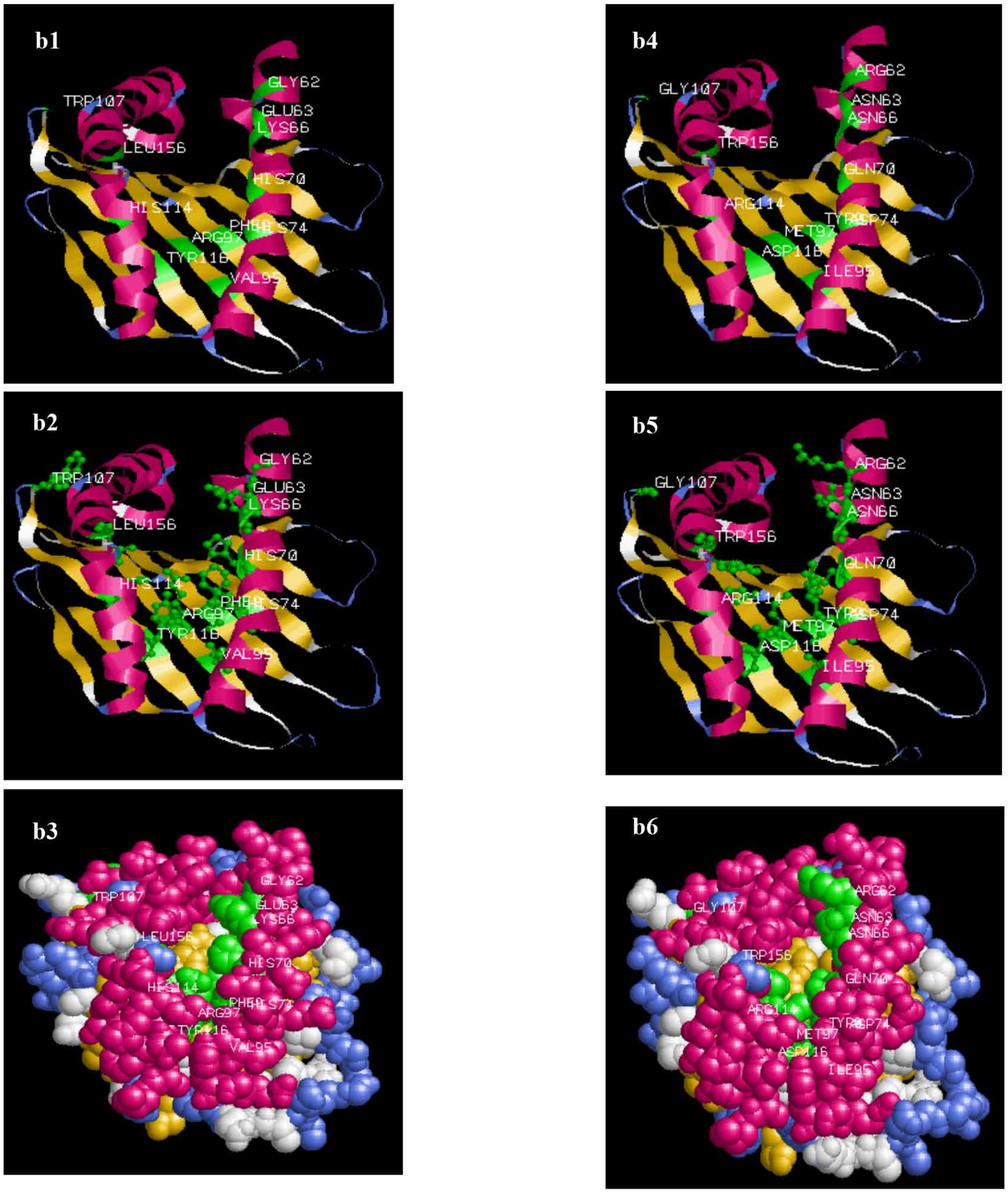

Supplementary Figure S5b: (*Continued*) Eight representative of three-dimensional structure modeling analysis for each mismatched HLA-A/-B/-DRB1/-DQB1 alleles on allo-HSCT recipient-donor pairs from either retrospective or prospective studies. In figure 5b. the display patterns are similar to figure 5a. The b1-b3 and b4-b6 represent three-dimensional structure modeling analysis of the a1 and a2 domains for HLA -A*02:01/A*68:01 of R-UPN07 donor-recipient pair. The amino acids 9, 62, 63, 66,70, 74, 95,97,107,114,116 and 156 are the different AARs between HLA-A*02:01 and A*68:01. From figure b1 to b6, it can be clearly observed that the structural differences between the mismatched AARs in peptide-binding sites or TCR interaction positions were far larger, especially at AARs 62,63,66,70 and 114. With a 0.4317Å of total revised RMSD value, grade IV aGVHD occurred in R-UPN07 after transplantation. (*Continued*)

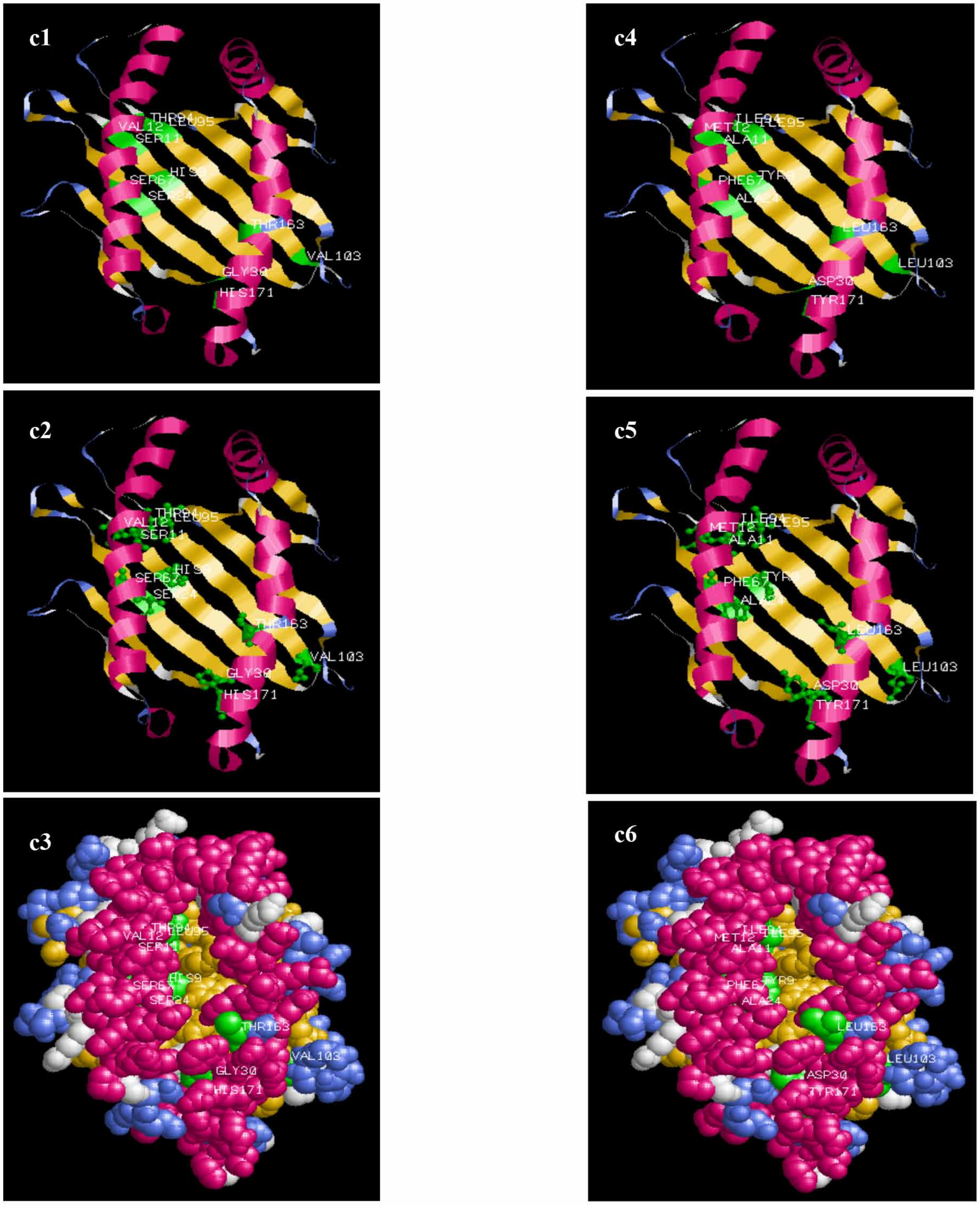

Supplementary Figure S5c: (*Continued*) Eight representative of three-dimensional structure modeling analysis for each mismatched HLA-A/-B/-DRB1/-DQB1 alleles on allo-HSCT recipient-donor pairs from either retrospective or prospective studies. In figure 5c. the display patterns are similar to figure 5a . The c1-c3 and c4-c6 represent three-dimensional structure modeling analysis of the a1 and a2 domains for HLA-B*18:01/B*35:01 of P-UPN06 donor-recipient pair. The amino acids 9,11,12,24,30,67,94, 95,103,163 and 171 are the different AARs between HLA-B*18:01/B*35:01. From figure c1 to c6, it can be clearly observed that the structural differences between the mismatched AARs in peptide-binding sites or TCR interaction positions were far smaller with only 0.1832Å of total revised RMSD value, therefore only grade I aGVHD occurred in P-UPN06 after transplantation. (*Continued*)

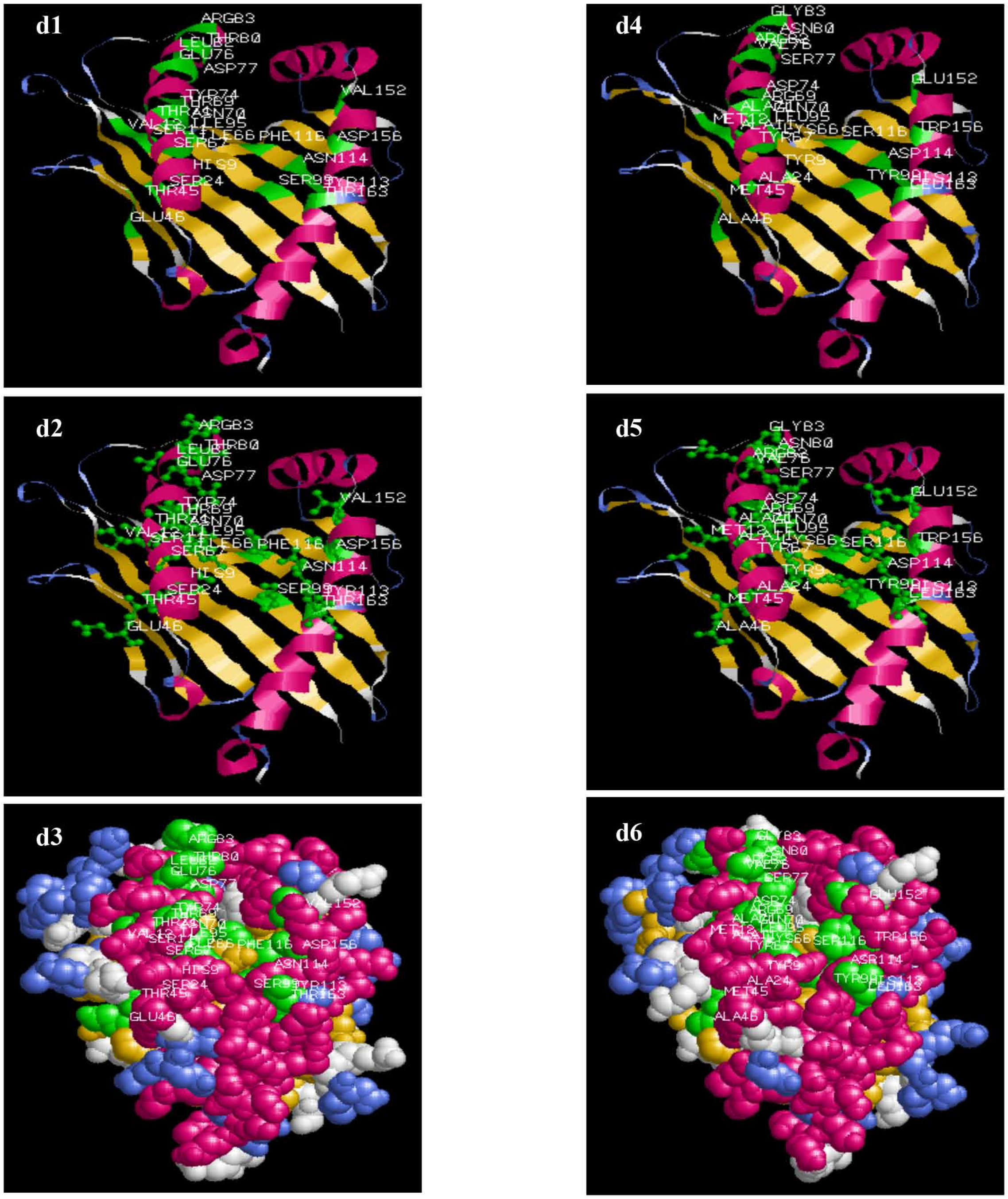

Supplementary Figure S5d: (*Continued*) Eight representative of three-dimensional structure modeling analysis for each mismatched HLA-A/-B/-DRB1/-DQB1 alleles on allo-HSCT recipient-donor pairs from either retrospective or prospective studies. In figure 5d. the display patterns are similar to figure 5a . The d1-d3 and d4-d6 represent three-dimensional structure modeling analysis of the a1 and a2 domains for HLA-B*37:01/B*46:01 of P-UPN15 donor-recipient pair. The amino acids 9,11,12,24,45,46,66,67, 69,70,71,74,76,77, 80,82,83,95,99,113,114,116,152, 156 and 163 are the different AARs between HLA-B*37:01/B*46:01. From figure d1 to d6, it can be clearly observed that the structural differences between the mismatched AARs in peptide-binding sites or TCR interaction positions were far larger, especially at AARs 66,67,69,70,74,76, 77,80,83,95,113,116 and 163. With a 0.8127 Å of total revised RMSD value, grade IV aGVHD occurred in P-UPN15 after transplantation. (*Continued*)

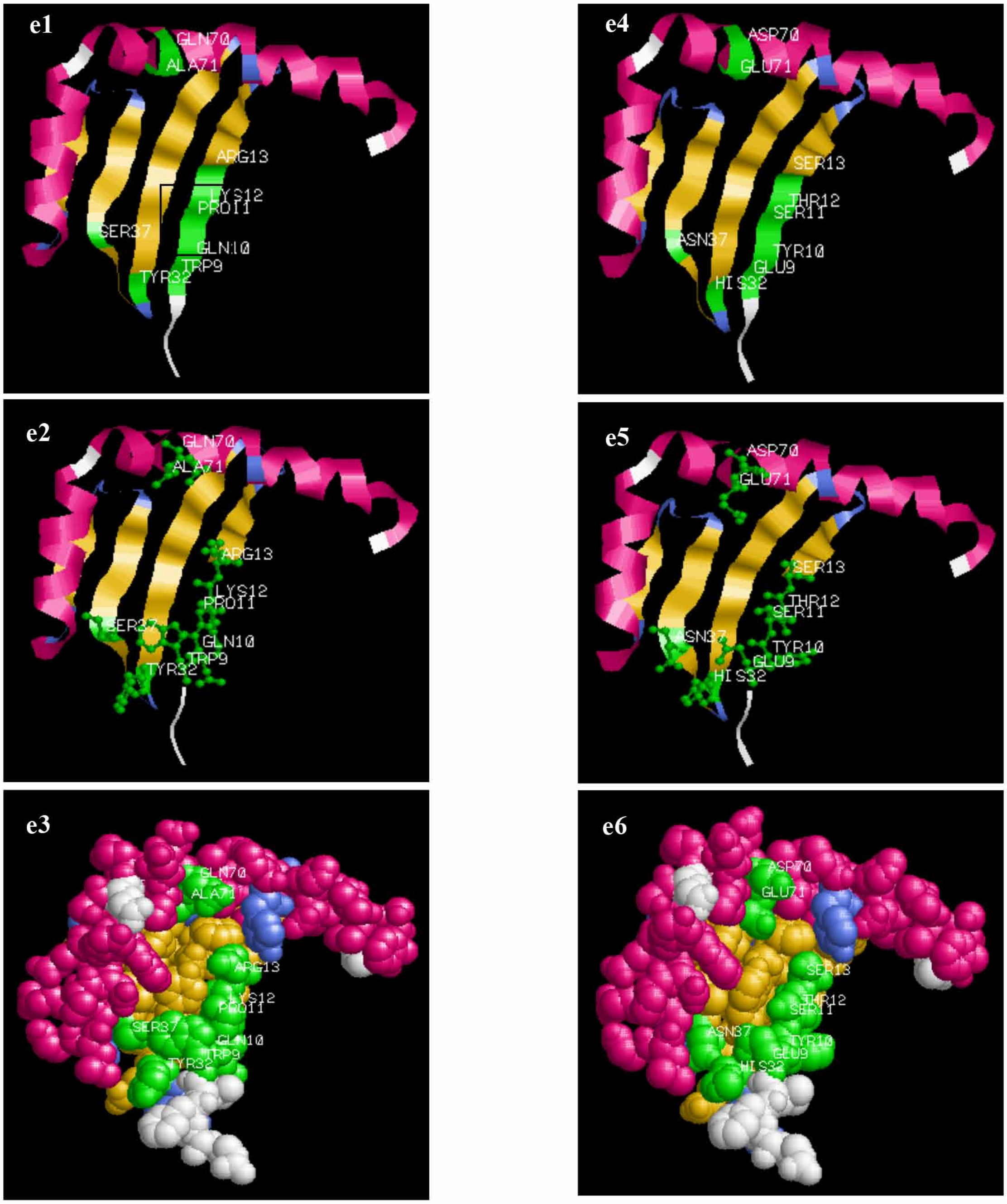

Supplementary Figure S5e: (*Continued*) Eight representative of three-dimensional structure modeling analysis for each mismatched HLA-A/-B/-DRB1/-DQB1 alleles on allo-HSCT recipient-donor pairs from either retrospective or prospective studies. In figure 5e. the display patterns are similar to figure 5a. The e1-e3 and e4-e6 represent three-dimensional structure modeling analysis of the a1 and a2 domains for HLA-DRB1*15:01/DRB1*13:01 of R-UPN01 donor-recipient pair. The amino acids 9, 10,11, 12,13,32,37,70 and 71 are the different AARs between HLA-DRB1*15:01 /DRB1*13:01. From figure e1 to e6, it can be clearly observed that the structural differences between the mismatched AARs in peptide-binding sites or TCR interaction positions were far smaller with only 0.2958Å of total revised RMSD value, therefore only grade I aGVHD occurred in R-UPN01 after transplantation. (*Continued*)

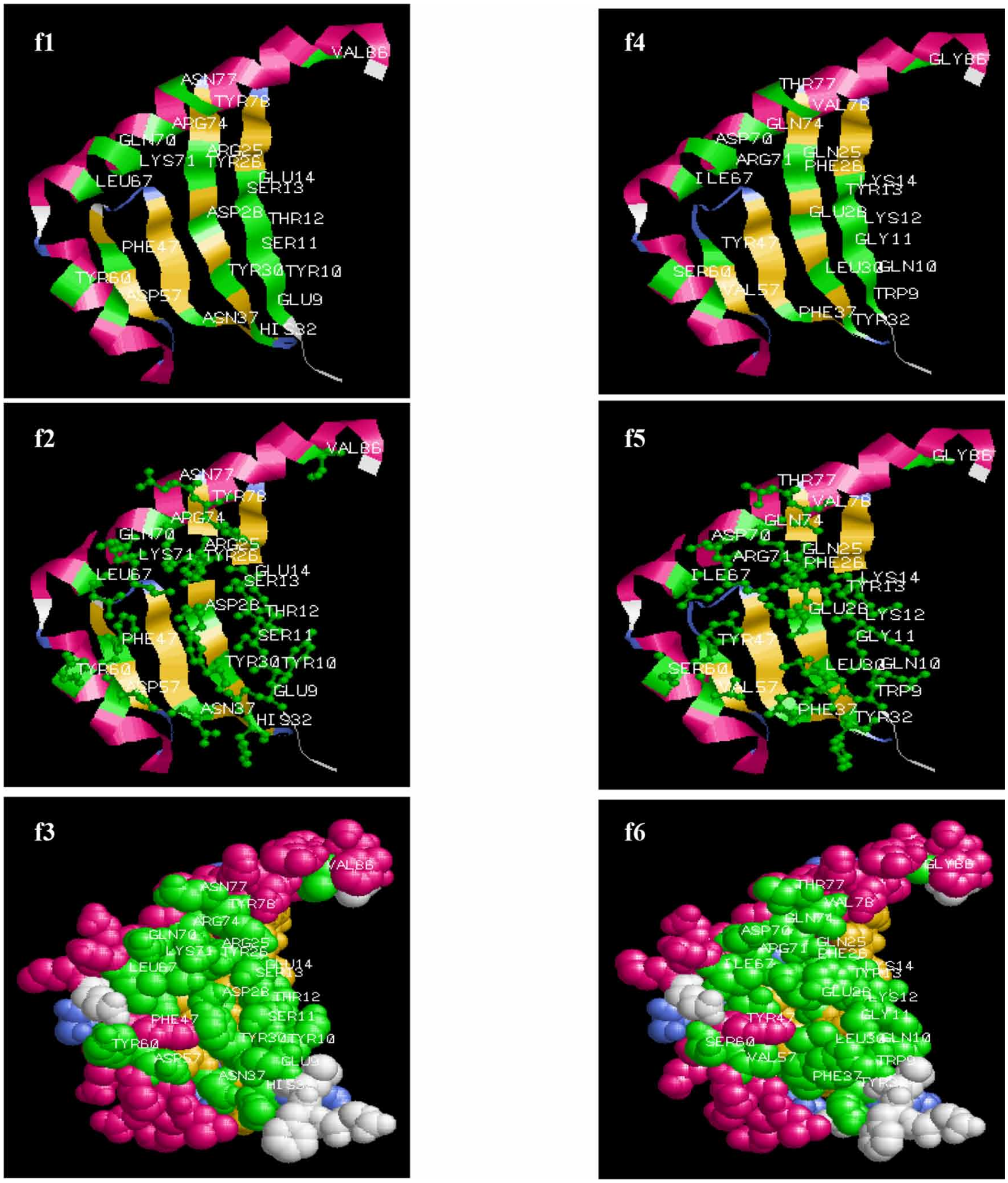

Supplementary Figure S5f: (*Continued*) Eight representative of three-dimensional structure modeling analysis for each mismatched HLA-A/-B/-DRB1/-DQB1 alleles on allo-HSCT recipient-donor pairs from either retrospective or prospective studies. In figure 5f. the display patterns are similar to figure 5a. The f1-f3 and f4-f6 represent three-dimensional structure modeling analysis of the a1 and a2 domains for HLA-DRB1*03:01/DRB1*07:01 of P-UPN18 donor-recipient pair. The amino acids 9,10,11, 12,13,14,25,26,28,30,32,37,47,57,60, 67,70,71,74,77,78 and 86 are the different AARs between HLA-DRB1*03:01/ DRB1*07:01. From figure f1 to f6, it can be clearly observed that the structural differences between the mismatched AARs in peptide-binding sites or TCR interaction positions were far larger, especially at AARs 37, 47, 57,60,67,70,71,74,77 and 78. With a 0.5978Å of total revised RMSD value, grade IV aGVHD occurred in P-UPN18 after transplantation. (*Continued*)

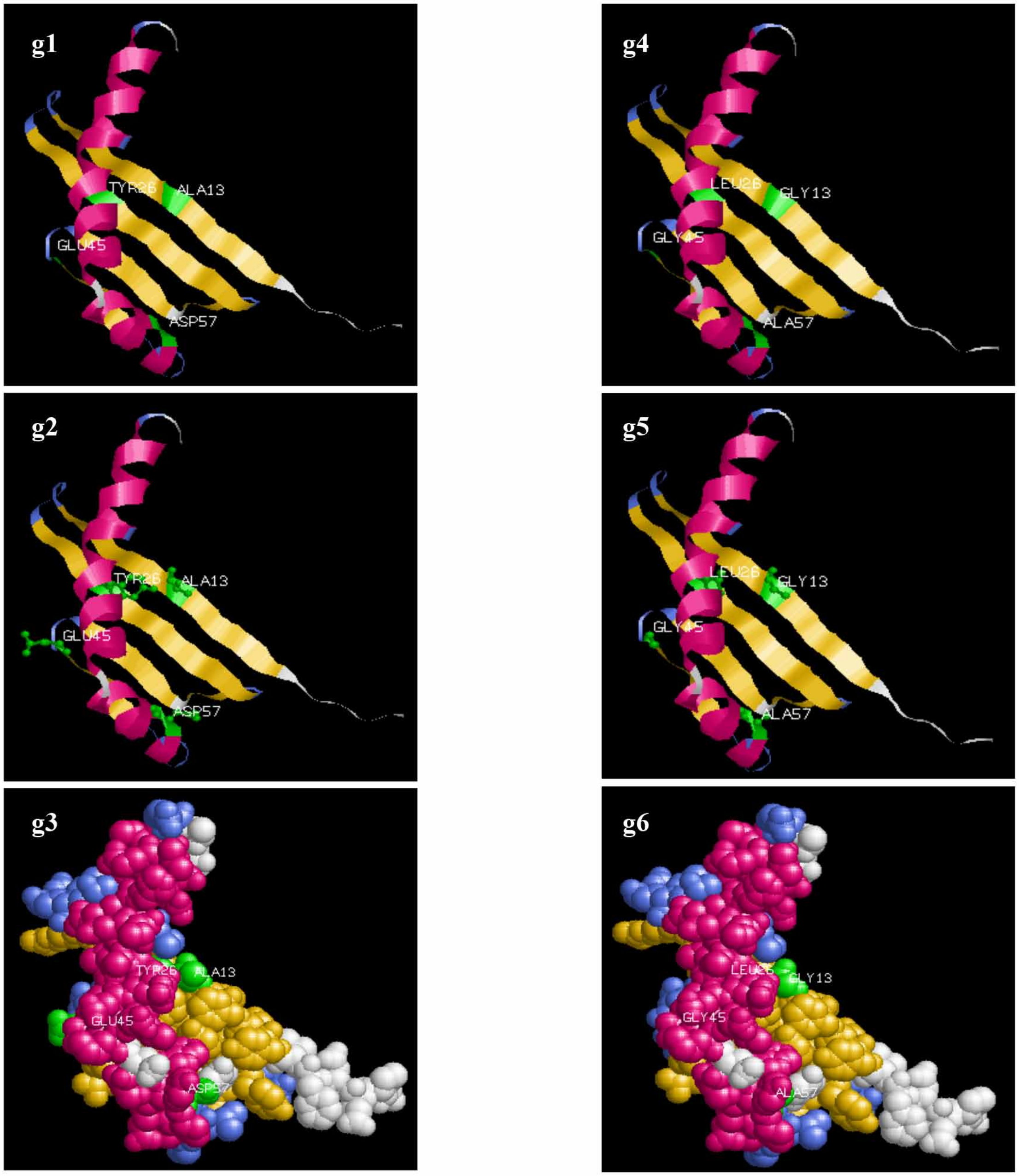

Supplementary Figure S5g: (*Continued*) Eight representative of three-dimensional structure modeling analysis for each mismatched HLA-A/-B/-DRB1/-DQB1 alleles on allo-HSCT recipient-donor pairs from either retrospective or prospective studies. In figure 5g. the display patterns are similar to figure 5a. The g1-g3 and g4-g6 represent three-dimensional structure modeling analysis of the a1 and a2 domains for HLA-DQB1*03:01/ DQB1*03:02 off R-UPN12 donor-recipient pair. The amino acids 13, 26, 45 and 57 are the different AARs between HLA-DRB1*15:01/DRB1*13:01. From figure g1 to g6, it can be clearly observed that the structural differences between the mismatched AARs in peptide-binding sites or TCR interaction positions were far smaller with only 0.2211Å of total revised RMSD value, therefore only grade I aGVHD occurred in R-UPN12 after transplantation. (*Continued*)

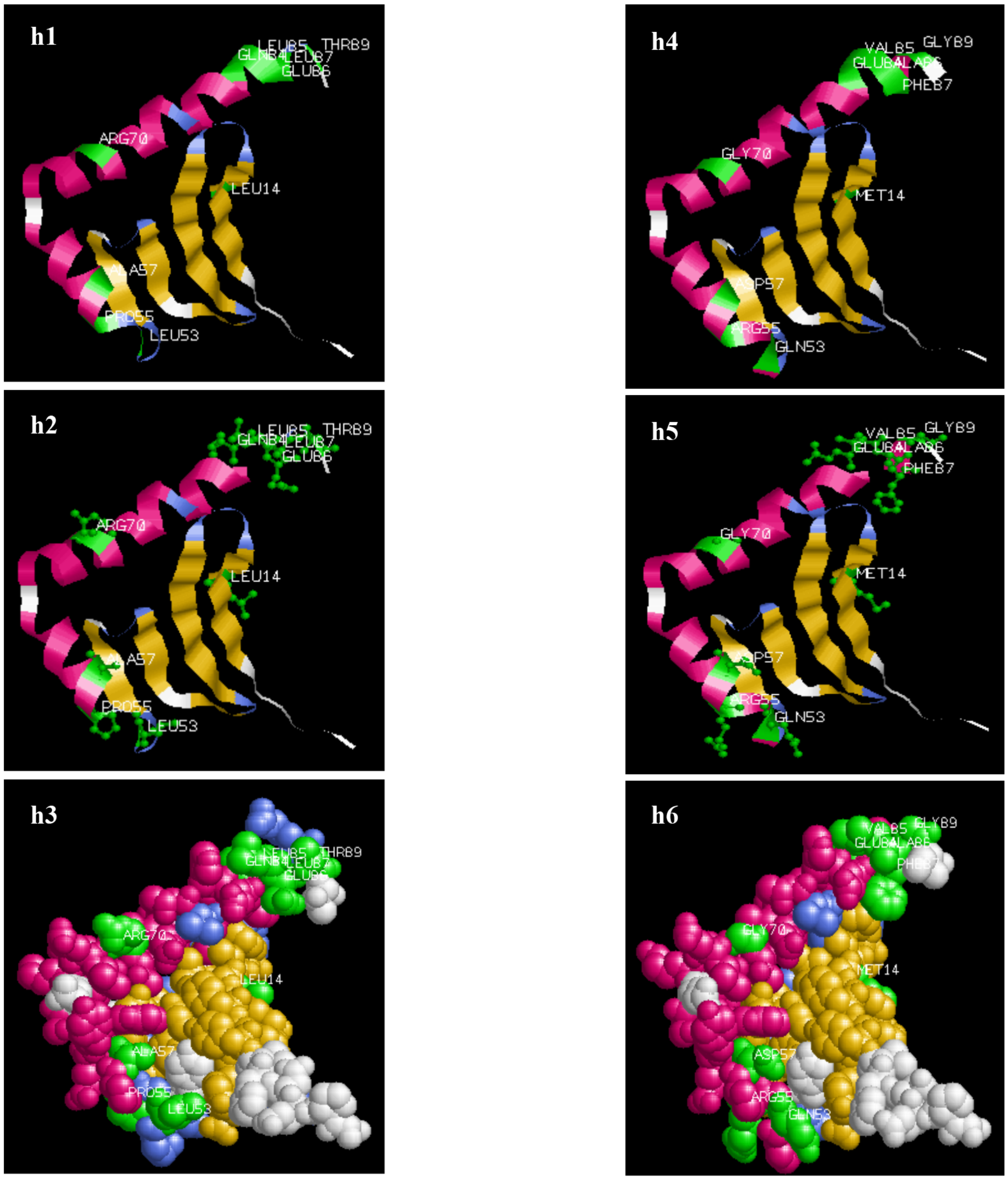

Supplementary Figure S5h: (*Continued*) Eight representative of three-dimensional structure modeling analysis for each mismatched HLA-A/-B/-DRB1/-DQB1 alleles on allo-HSCT recipient-donor pairs from either retrospective or prospective studies. In figure 5h. the display patterns are similar to figure 5a. The h1-h3 and h4-h6 represent three-dimensional structure modeling analysis of the a1 and a2 domains for HLA-DQB1*03:11/ DQB1*06:11 of R-UPN10 donor-recipient pair. The amino acids 14, 53,55,57,70,84,85,86, 87 and 89 are the different AARs between HLA-DQB1*03:11/DQB1*06:11. From figure h1 to h6, it can be clearly observed that the structural differences between the mismatched AARs in peptide-binding sites or TCR interaction positions were far larger. All of AARs were located at the sidewalls and the tops of antigen-binding groove except residue 14 which was at the bottom. With a 0.6333Å of total revised RMSD value, grade IV aGVHD occurred in R-UPN10 after transplantation.

Supplementary Table S1: The defined HLA unacceptable/impermissible mismatching allele pairs based on revised RMSD at HLA-A*02/-B*15/-DRB1*04 allele groups respectively

Alleles[+]	HLA unacceptable/impermissible mismatching allele pairs in HLA-A*02/-B*15/-DRB1*04 allele groups
	**HLA-A*02 allele group**
A*02:01	A*02:08
A*02:08	A*02:09, A*02:13, A*02:14, A*02:26, A*02:47, A*02:63, A*02:65
A*02:11	A*02:47
A*02:13	A*02:21, A*02:28, A*02:29, A*02:51, A*02:52, A*02:61, A*02:72
A*02:21	A*02:47, A*02:63, A*02:65
A*02:28	A*02:47, A*02:63, A*02:65
A*02:29	A*02:47, A*02:63, A*02:65
A*02:34	A*02:47
A*02:35	A*02:47
A*02:38	A*02:65
A*02:39	A*02:47
A*02:47	A*02:51, A*02:52, A*02:57, A*02:60, A*02:61, A*02:69, A*02:72, A*02:78
A*02:51	A*02:63, A*02:65
A*02:52	A*02:63, A*02:65
A*02:61	A*02:63, A*02:65
A*02:63	A*02:72
A*02:65	A*02:72
A*02:66–1	**............**(get more detailed by HLA-TDSM system)
	**HLA-DRB1*04 allele group**
DRB1*04:01–19	DRB1*04:20
DRB1*04:20	RB1*04:21- DRB1*04:53
	...........(get more detailed by HLA-TDSM system)
B*15:01	B*15:13, B*15:16–18, B*15:20, B*15:23, B*15:24, B*15:29, B*15:36, B*15:37, B*15:51, B*15:52, B*15:62, B*15:67, B*15:72, B*15:80, B*15:83, B*15:87–89, B*15:93, B*15:95, B*15:99
B*15:02	B*15:13, B*15:16, B*15:17, B*15:23, B*15:24, B*15:29, B*15:36, B*15:37, B*15:51, B*15:52, B*15:62, B*15:67, B*15:80, B*15:83, B*15:87, B*15:89, B*15:93, B*15:95
B*15:03	B*15:13, B*15:16–18, B*15:20, B*15:23, B*15:24, B*15:29, B*15:36, B*15:37, B*15:51, B*15:52, B*15:62, B*15:67, B*15:72, B*15:80, B*15:83, B*15:87–89, B*15:93, B*15:95, B*15:99
B*15:04	B*15:13, B*15:16, B*15:17, B*15:23, B*15:24, B*15:29, B*15:36, B*15:37, B*15:51, B*15:62, B*15:67, B*15:83, B*15:87, B*15:89, B*15:93, B*15:95
B*15:05	B*15:13, B*15:16, B*15:17, B*15:23, B*15:24, B*15:29, B*15:36, B*15:37, B*15:51, B*15:52, B*15:62, B*15:67, B*15:80, B*15:83, B*15:87, B*15:89, B*15:93, B*15:95
B*15:06	B*15:13, B*15:16, B*15:17, B*15:23, B*15:24, B*15:29, B*15:36, B*15:37, B*15:51, B*15:62, B*15:67, B*15:83, B*15:87, B*15:89, B*15:93, B*15:95
B*15:07	B*15:13, B*15:16–18, B*15:20, B*15:23, B*15:24, B*15:29, B*15:36, B*15:37, B*15:51, B*15:52, B*15:62, B*15:67, B*15:80, B*15:83, B*15:87–89, B*15:93, B*15:95, B*15:99

Alleles+	HLA unacceptable/impermissible mismatching allele pairs in HLA-A*02/-B*15/-DRB1*04 allele groups
B*15:08	B*15:13, B*15:16, B*15:17, B*15:23, B*15:24, B*15:29, B*15:36, B*15:37, B*15:51, B*15:52, B*15:62, B*15:67, B*15:80, B*15:83, B*15:87, B*15:89, B*15:93, B*15:95
B*15:09	B*15:13, B*15:16, B*15:17, B*15:23, B*15:24, B*15:29, B*15:36, B*15:37, B*15:51, B*15:62, B*15:67, B*15:83, B*15:87, B*15:89, B*15:93, B*15:95
B*15:10	B*15:13, B*15:16, B*15:17, B*15:23, B*15:24, B*15:29, B*15:36, B*15:37, B*15:51, B*15:62, B*15:67, B*15:83, B*15:87, B*15:89, B*15:93, B*15:95
B*15:11	B*15:13, B*15:16, B*15:17, B*15:23, B*15:24, B*15:29, B*15:36, B*15:37, B*15:51, B*15:52, B*15:62, B*15:67, B*15:80, B*15:83, B*15:87, B*15:89, B*15:93, B*15:95
B*15:12	B*15:13, B*15:16–18, B*15:23, B*15:24, B*15:29, B*15:36, B*15:37, B*15:51, B*15:52, B*15:62, B*15:67, B*15:80, B*15:83, B*15:87–89, B*15:93, B*15:95
B*15:13	B*15:14, B*15:15, B*15:18–21, B*15:25, B*15:27–29, B*15:31–35, B*15:37–40, B*15:42, B*15:44–58, B*15:60–66, B*15:68–78, B*15:80–86, B*15:88–93, B*15:96–99
B*15:14–13	**............**(get more detailed by HLA-TDSM system)

+ HLA-A*02/-B*15/-DRB1*04 allele groups include at least 392,264 and 133 alleles respectively, all of whom are the most polymorphic in their respective locus. For these reasons, we selected HLA-A*02/-B*15/-DRB1*04 allele groups as the representative of unacceptable/impermissible mismatching allele pairs from HLA allele groups. The matched criteria of the revised RMSD value are □0.2Å for HLA-A*02/-B*15 allele groups and □0.1Å for HLA-DRB1*04 allele group. The limited layout sake this table shows only partial results.

Supplementary Table S2: Experimental design for allo-reactivity patterns induced by different CTLs clones

PBMCInducing MHC-Pep	Exp A				Exp B				Exp C			
	**15:02**	**15:18**	**35:03**	**44:03**	**15:02**	**15:18**	**35:03**	**44:03**	**15:02**	**15:18**	**35:03**	**44:03**
Target cells	15:02	NT	15:02	15:02	15:02	15:02	15:02	15:02	15:02	15:02	15:02	15:02
	15:18	NT	15:18	15:18	15:18	15:18	15:18	15:18	15:18	15:18	15:18	15:18
	35:03	NT	35:03	35:03	35:03	35:03	35:03	35:03	35:03	35:03	35:03	35:03
	44:03	NT	44:03	44:03	44:03	44:03	44:03	44:03	44:03	44:03	44:03	44:03

Exp A, Exp B and Exp C:T cells of peripheral blood from 3 normal volunteers. Inducing MHC-Pep: stable transfected Hmy2.CIR cell by HLA-B molecules pulsed with Epstein-Barr virus nonameric peptide (EBNA3).

Supplementary Table S3: Allogeneic cross-reaction patterns by the induced antigen-specific alloreactive CTLs *in vitro*

CTLs induced by	T cell	Proliferation	Cytotoxicity
Hmy-B*15:02	ExpA	B*15:02 specific.	B*15:02 specific
	ExpB	B*15:02, B*15:18	B*15:02, B*15:18, B*35:03, B*44:03
	ExpC	B*15:02, B*15:18, B*35:03	B*15:02, B*15:18, B*35:03
Hmy-B*15:18	ExpA		
	ExpB	B*15:02, B*15:18	B*15:02, B*15:18, B*35:03, B*44:03
	ExpC	B*15:18 specific	B*15:02, B*15:18
Hmy-B*35:03	ExpA	B*15:02, B*35:03	
	ExpB	B*15:18, B*35:03	B*15:18, B*35:03 cross reaction.
	ExpC	B*15:02, B*15:18, B*35:03	B*15:02, B*15:18, B*35:03
Hmy-B*44:03	ExpA	non-specific	
	ExpB	non-specific.	B*15:18, B*35:03, B*44:03
	ExpC	B*15:18, B*44:03	B*15:02, B*35:03, B*44:03

In our study, transfected Hmy2.CIR cells pulsed with a nonameric consensus peptide (EBNA3) were used as target cells and inducing cells in the vitro experiments, and HLA antigens were the only variable factors in TCR-MHC-Pep. And then the cross-reaction pattern of induced CTLs could reflect the function differences of HLA antigens indirectly. Proliferation inhibition or cytotoxicity of the induced CTLs origin from Exp A were low in general, while HLA-B*15:02 induced CTLs identified specifically and HLA-B*35:03 induced CTLs exhibited cross-reaction obviously. Cross-reaction were existed commonly of the induced CTLs origin from Exp B, reflected as HLA-B*15:02 induced CTLs cross-reaction to HLA-B*35:05, HLA-B*35:05 induced CTLs cross-reaction to HLA-B*15:02 and HLA-B*15:18. As to the induced CTLs origin from Exp C, cross-reaction were displayed as HLA-B*35:05 induced CTLs cross-reaction to HLA-B*15:02 and HLA-B*15:18.

Supplementary Table S4: The sequence comparison among HLA-B*15:02, -B*15:18, -B*35:03 and -B*44:03 alleles

HLA alleles	Position of amino acid residue and antigen binding pocket																	
	24	32	41	45	46	63	67	77	80	81	82	83	94	95	103	113	116	152	167
	B			B		AB	B	F	F	F							F	E	A
B*15:02	A	Q	A	M	A	N	S	S	N	L	R	G	I	I	V	Y	S	E	W
B*15:18	S	Q	A	E	E	N	C	S	N	L	R	G	T	L	V	H	S	E	W
B*35:03	A	Q	A	T	E	N	F	S	N	L	R	G	I	I	L	H	F	V	W
B*44:03	T	L	T	K	E	E	S	N	T	A	L	R	I	I	V	Y	D	V	S

1 http://www.ebi.ac.uk/ipd/imgt/hla/
2 Robinson J, Mistry K, McWilliam H, Lopez R, Parham P, Marsh SG. The IMGT/HLA Database. Nucleic Acids Research. 2011; Suppl 1:D1171-6.
3 Saper MA, Bjorkman PJ, Wiley DC. Refined structure of the human histocompatibility antigen HLA-A2 at 2.6A resolution. J Mol Biol. 1991;219: 277-319.
4 Garboczi DN, Ghosh P, Utz U, Fan QR, Biddison WE, Wiley DC. Structure of the complex between human T-cell receptor, viral peptide and HLA-A2. Nature. 1996; 384:134-41.
